# TMED10 mediates the trafficking of insulin-like growth factor 2 along the secretory pathway for myoblast differentiation

**DOI:** 10.1073/pnas.2215285120

**Published:** 2023-11-06

**Authors:** Tiantian Li, Feng Yang, Youshan Heng, Shaopu Zhou, Gang Wang, Jianying Wang, Jinhui Wang, Xianwei Chen, Zhong-Ping Yao, Zhenguo Wu, Yusong Guo

**Affiliations:** ^a^Division of Life Science and State Key Laboratory of Molecular Neuroscience, The Hong Kong University of Science and Technology, Hong Kong, China; ^b^State Key Laboratory of Chemical Biology and Drug Discovery, Research Institute for Future Food, Research Centre for Chinese Medicine Innovation, and Department of Applied Biology and Chemical Technology, The Hong Kong Polytechnic University, Hong Kong, China; ^c^State Key Laboratory of Chinese Medicine and Molecular Pharmacology (Incubation) and Shenzhen Key Laboratory of Food Biological Safety Control, Hong Kong Polytechnic University, Shenzhen Research Institute, Shenzhen 518057, China; ^d^Hong Kong University of Science and Technology, Shenzhen Research Institute, Shenzhen 518057, China; ^e^Thrust of Bioscience and Biomedical Engineering, Hong Kong University of Science and Technology, Guangzhou 511453, China

**Keywords:** secretion, IGF2, TMED10, sorting, COPII

## Abstract

The insulin-like growth factor 2 (IGF2) plays critical roles in cell proliferation, migration, differentiation, and survival. Despite its importance, the molecular mechanisms mediating the trafficking of IGF2 along the secretory pathway remain unclear. Here, we utilized a Retention Using Selective Hook system to analyze molecular mechanisms that regulate the secretion of IGF2. We found that a type I transmembrane protein, TMED10, is essential for the secretion of IGF2 and for differentiation of mouse myoblast C2C12 cells. Further analyses indicate that the residues 112-140 in IGF2 are important for the secretion of IGF2 and these residues directly interact with the GOLD domain of TMED10. We then reconstituted the release of IGF2 into COPII vesicles. This assay suggests that TMED10 mediates the packaging of IGF2 into COPII vesicles to be efficiently delivered to the Golgi. Moreover, TMED10 also mediates ER export of TGN-localized cargo receptor, sortilin, which subsequently mediates TGN export of IGF2. These analyses indicate that TMED10 is critical for IGF2 secretion by directly regulating ER export and indirectly regulating TGN export of IGF2, providing insights into trafficking of IGF2 for myoblast differentiation.

## Significance

Insulin-like growth factor 2 (IGF2) is a key regulator of skeletal myogenesis during development. Currently, mechanisms regulating IGF2 expression and the signal transduction pathway induced by IGF2 have been extensively investigated. However, how IGF2 proteins, after synthesized from ribosomes, are secreted to perform their functions remains elusive. We demonstrate that a cargo receptor, transmembrane emp24 domain-containing protein 10 (TMED10), promotes endoplasmic reticulum (ER) export of IGF2 via recognizing an export signal on IGF2. Moreover, TMED10 also mediates ER export of sortilin, which is important to regulate the export of IGF2 from the *trans-* Golgi network. These analyses provide insight into the mechanisms regulating IGF2 secretion, filling an important gap in our understanding of IGF2 from its expression to its function.

Insulin-like growth factor 2 (IGF2) is involved in various physiological activities, especially for skeletal myogenesis (1). It is an embryonic regulator of myogenesis and an autocrine factor that promotes myoblast differentiation in vitro (2–4). Knockdown of IGF2 in mouse muscle myoblasts impaired the differentiation progress, demonstrating that IGF2 regulates muscle stem cell differentiation (3). Despite its importance, molecular mechanisms that mediate the secretion of IGF2 proteins from producing cells are poorly understood.

After synthesized from ribosomes, IGF2 needs to be delivered along the secretory transport pathway to perform its physiological functions. IGF2 is first synthesized as a precursor hormone containing 180 amino acids. After being imported into the ER, an N-terminal signal peptide is cleaved, generating pro-IGF2 (IGF2^25-180^). The correctly folded pro-IGF2 proteins are then packaged into transport vesicles to be delivered to the Golgi apparatus. At the Golgi, pro-IGF2 undergoes O-glycosylation modifications and endoproteolysis, generating IGF2 peptides IGF2^25-128^, IGF2^25-111^, and the mature IGF2 (IGF2^25-91^) (5).

Coat protein complex II (COPII) is the key player that regulates the packaging of cargo proteins into vesicles at the ER. In the conventional secretory transport pathway, soluble cargo proteins in the lumen of the ER cannot be directly recognized by the COPII coat; instead, these cytosolic proteins are thought to be transported under the recognition of transmembrane cargo receptors (6). ERIGIC53 is a major cargo receptor that recruits a variety of soluble cargo proteins to COPII vesicles in mammals (6). In addition, the p24 proteins play crucial roles in ER-Golgi bidirectional transport. Some p24 family members function as cargo receptors to regulate ER export of specific GPI-anchored proteins and autotaxin in mammalian cells (6, 7) and to mediate secretion of Wnt proteins in Drosophila (8, 9). Soluble cargo proteins are also proposed to enter the nascent COPII vesicles by default, a process referred to as bulk flow (10). It is currently unknown whether the trafficking of IGF2 is passively regulated by bulk flow or is actively mediated by cargo receptors.

The *trans*-Golgi network (TGN) is another important station in the secretory transport pathway. At the TGN, various cargo adaptors and receptors have been shown to capture cargo molecules into nascent vesicles (11). Sortilin is one of the Golgi-localized cargo receptors that is crucial for the sorting of numerous proteins in the anterograde and retrograde pathways and is broadly involved in multiple physiological activities, including lipid metabolism, neuronal development, immune system, myogenesis and diabetes (12–16). Particularly, the noncoding genetic variants at a locus near gene encoding sortilin were significantly associated with LDL cholesterol and coronary artery disease in humans (17, 18). Further analysis indicates that sortilin interacts with apolipoprotein B100 (ApoB100) and modulates the secretion of ApoB100-containing lipoproteins in hepatocytes, but whether sortilin up-regulates or down-regulates lipoprotein secretion remains controversial (19, 20). The cargo receptors that deliver IGF2 from the TGN to the cell surface still remain unclear.

Here, we analyzed IGF2 intracellular transport utilizing the Retention Using Selective Hook (RUSH) assay. We also reconstituted the release of IGF2 into COPII vesicles through an in vitro vesicle formation assay to quantify the efficiency of cargo packaging. Through these approaches, we found that a p24 family protein, TMED10, functions as a cargo receptor to mediate ER export of IGF2 for myoblast differentiation. Moreover, we found that TMED10 regulates ER export of sortilin, and sortilin is important for TGN export of IGF2. These studies shed light on molecular mechanisms regulating IGF2 secretion along the secretory pathway for muscle stem cell differentiation.

## Results

### TMED10 Mediates ER-to-Golgi Trafficking and the Secretion of IGF2.

**Fig. 1.**
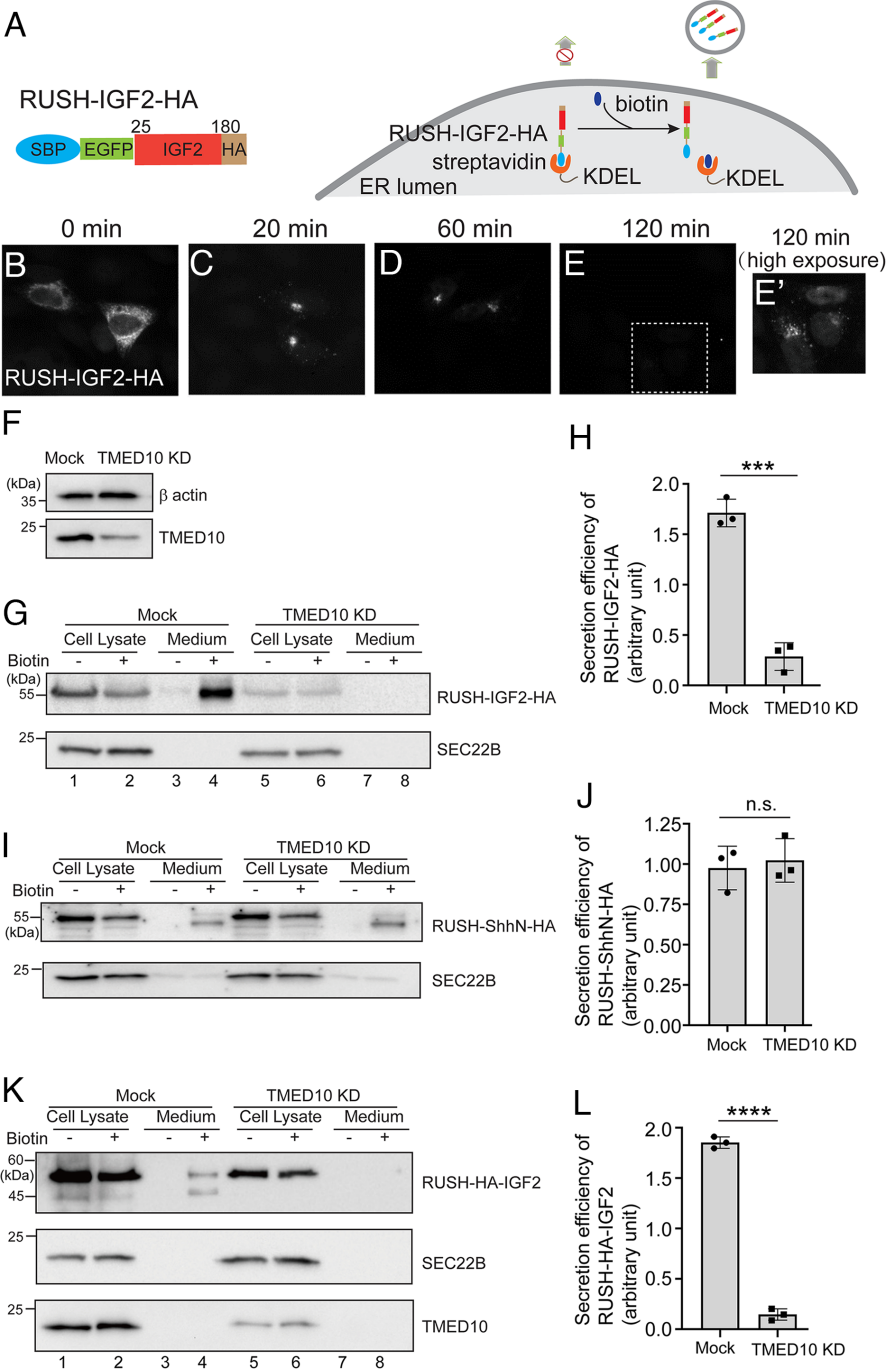
Knockdown of TMED10 causes defects in secretion of IGF2. (*A*) A diagram demonstrating the design of the RUSH-IGF2-HA construct and the RUSH assay. (*B*–*E*′) HeLa cells were transfected with plasmids encoding Str-KDEL and full-length RUSH-IGF2-HA. Day 1 after transfection, cells were preincubated with cycloheximide for 2 h. Then, the localization of RUSH-IGF2-HA was analyzed after incubating with biotin and cycloheximide for the indicated time. The view of the indicated area in panel *E* at a higher exposure was shown in panel *E*′. (*F*) HeLa cells were transfected with control siRNA or siRNA against TMED10. Day 2 after transfection, the level of TMED10 and β actin in cell lysates was analyzed by immunoblot. (*G*) Day 1 after transfection with siRNAs, cells were retransfected with plasmids encoding Str-KDEL and RUSH-IGF2-HA. On day 3 after knockdown, cells were preincubated with cycloheximide for 2 h. Then, cells were incubated with biotin and cycloheximide for 2 h. After biotin incubation, the level of RUSH-IGF2-HA in the medium and in cell lysates was analyzed by immunoblot. (*H*) Quantification of the abundance of secreted IGF2 normalized to the abundance detected in the cell lysate group in the absence of biotin (mean ± SD; *n* = 3). (*I*) Day 1 after transfection with siRNAs, cells were retransfected with plasmids encoding Str-KDEL and RUSH-ShhN-HA. On day 3 after knockdown, cells were preincubated with cycloheximide for 2 h. Then cells were incubated with biotin and cycloheximide for 2 h. After biotin incubation, the level of RUSH-ShhN-HA in the medium and in cell lysates was analyzed by immunoblot. (*J*) Quantification of the abundance of secreted ShhN normalized to the abundance detected in the cell lysate group in the absence of biotin (mean ± SD; *n* = 3). (*K*) Day 1 after transfection with siRNAs, cells were transiently transfected RUSH-HA-IGF2. On day 3 after knockdown, cells were preincubated with cycloheximide for 2 h. Then, cells were incubated with biotin and cycloheximide for 2 h. After biotin incubation, the abundance of RUSH-HA-IGF2 in the medium and in cell lysates was analyzed by immunoblot. (*L*) Quantification of the abundance of secreted IGF2 normalized to the abundance detected in the cell lysate in the absence of biotin (mean ± SD; *n* = 3). In each experimental group of each replicated experiment, the value was normalized to the average value of the Mock group and the TMED10 KD group (*H*, *J*, and *L*). ***P < 0.001; ****P < 0.0001; n.s., not significant.

We have recently performed coimmunoprecipitation (co-IP) experiments to reveal proteins that interact with the HA-tagged N-terminal fragment of sonic hedgehog (ShhN-HA) or HA-tagged IGF2 (IGF2-HA) (21). Label-free quantitative mass spectrometry analysis of the immunoprecipitated proteins identified a transmembrane protein, TMED10, which binds stronger to IGF2-HA than to ShhN-HA (21). TMED10 is an ER- and Golgi-localized transmembrane protein that belongs to the p24 family. To test whether TMED10 is important for IGF2 secretion, we utilized a RUSH transport assay (21–23). In this assay, HeLa cells were transfected with plasmids encoding human IGF2 (aa: 25-180, removed signal peptide) tagged with EGFP, the streptavidin binding peptide (SBP) and an HA tag at the C terminus (referred to as RUSH-IGF2-HA or RUSH-IGF2^25-180^-HA) (Fig. 1*A*). This plasmid also encodes streptavidin fused to a C-terminal ER retention signal (Lys-Asp-Glu-Leu; Str-KDEL). Due to the binding between streptavidin and SBP, RUSH-IGF2-HA was retained at the ER upon expression (Fig. 1*B*, 0 min). It is noted that the RUSH system does not halt ER export but rather creates an imbalance, favoring retrieval over export. Consequently, most of the cargo remains at the ER in the absence of biotin. When cells were incubated with biotin, SBP was uncoupled with streptavidin, thereby releasing RUSH-IGF2-HA from the ER retrieval process (Fig. 1 *C*–*E*′). Over 80% of cells showed Golgi-localized IGF2 when the cells were incubated with biotin for 20 min (Fig. 1*C*). After biotin treatment for 120 min, the signal of RUSH-IGF2-HA was greatly reduced (Fig. 1 *E* and *E*′), suggesting that IGF2 was secreted out of the cells or delivered to lysosomes for degradation.

We measured the efficiency of ER-to-Golgi trafficking of RUSH-IGF2-HA by quantifying the percentage of cells showing juxta-nuclear located RUSH-IGF2-HA following biotin treatment. This method does not differentiate between cells in which RUSH cargo proteins are partly located in the ER and partly in the perinuclear Golgi region, and those in which all RUSH cargo proteins are located in the Golgi. Despite this limitation, this quantification approach has been efficiently employed previously to measure the efficiency of ER-to-Golgi trafficking of RUSH cargo proteins (21, 24, 25). To test whether the trafficking of the cargo protein reaches saturation, we performed experiments to analyze the trafficking of RUSH-IGF2-HA at varying expression levels. For this, we transfected HeLa cells with different concentrations (0.8 μg/mL, 1.6 μg/mL, 3.2 μg/mL) of plasmids encoding RUSH-IGF2-HA. 1.6 μg/mL is the standard concentration we used in our study. We observed that as the DNA concentration for transfection increased, so did the expression level of RUSH-IGF2-HA in the cell lysates (*SI Appendix*, Fig. S1*A*). Across all conditions and expression levels, RUSH-IGF2-HA were detected in the juxta-nuclear Golgi area in around 80% of cells 20 min after biotin treatment (*SI Appendix*, Fig. S1*B*). Notably, the percentage showed no significant difference across all conditions. Furthermore, we identified a positive correlation between the expression level of RUSH-IGF2-HA and the abundance of secreted RUSH-IGF2-HA, two hours after biotin treatment (*SI Appendix*, Fig. S1*C*). A similar positive correlation was also found between the abundance of secreted ShhN-HA and the expression level of ShhN-HA (21). These findings suggest that these cargo molecules, irrespective of their expression levels, can be efficiently transported to the Golgi and secreted, without reaching saturation. One explanation for the ability of a limited number of cargo receptors to mediate trafficking of a large volume of clients is the recycling mechanism of these cargo receptors. After delivering their clients to the destination, the cargo receptors disassociate and return to their original location to initiate the next round of trafficking.

We then performed an siRNA knockdown (KD) experiment to test whether TMED10 is required for IGF2 secretion. Given that the abundance of secreted proteins generally correlates positively with their expression levels, we normalized the quantity of secreted IGF2 against the abundance found in the cell lysate in the absence of biotin. This normalized value serves as an indicator of the efficiency of IGF2 secretion. The expression of TMED10 was greatly reduced in HeLa cells transfected with siRNA against TMED10 (Fig. 1*F*). Remarkably, the secretion efficiency of RUSH-IGF2-HA was significantly decreased in TMED10 KD cells (Fig. 1*G*, compare lanes 4 and 8, and Fig. 1*H*), indicating that TMED10 plays an important role in IGF2 secretion. In contrast, the secretion of another protein, ShhN, was not affected in TMED10 KD cells (Fig. 1 *I* and *J*), demonstrating that TMED10 is a specific regulator for IGF2 rather than a common regulator.

We then generated TMED10 knockout (KO) HeLa cells to test the effect of depleting TMED10 on IGF2 secretion. Western blot analysis indicates that TMED10 was completely depleted in TMED10 KO HeLa cells (*SI Appendix*, Fig. S2*A*). The efficiency of RUSH-IGF2-HA secretion in TMED10 KO cells was also significantly reduced (*SI Appendix*, Fig. S2*B*, compare lanes 4 and 8, and *SI Appendix*, Fig. S2*C*), demonstrating that TMED10 is essential for IGF2 secretion.

We noticed that the size of the secreted RUSH-IGF2-HA detected by anti-HA antibodies is similar to the size of RUSH-IGF2-HA in cell lysates, suggesting that the secreted RUSH-IGF2-HA we detected is not the cleaved form. A possible explanation is that we used antibodies detecting the HA tag at the C terminus of IGF2 for the immunoblot analyses. We repeated this assay by using N-terminal HA-tagged RUSH-IGF2 (referred to as RUSH-HA-IGF2) to monitor the secretion of both big IGF2 and mature IGF2. We detected two bands in the medium group, and their molecular weights match the predicted molecular weights of the pro-IGF2 and mature IGF2 (Fig. 1*K*, lane 4). Consistent with the previous analyses, the secretion of both pro-IGF2 and mature IGF2 was reduced after TMED10 KD (Fig. 1*K*, compare lanes 4 and 8, and Fig. 1*L*).

We found that the abundance of RUSH-IGF2-HA in cell lysates from TMED10 KD or KO groups was decreased after biotin treatment (Fig. 1*K*, lanes 5–6, *SI Appendix*, Fig. S2*B*, lanes 5–6). We hypothesize that some of the RUSH-IGF2-HA have been degraded rather than secreted. To test this, we performed the RUSH assay using TMED10 KO cells and treated the cells with the lysosomal inhibitor (bafilomycin A1), or the proteasome inhibitor (MG132) in the presence of biotin. We found that bafilomycin A1 treatment enhanced the abundance of RUSH-IGF2-HA in cell lysates after biotin treatment, whereas MG132 treatment only showed a modest effect (*SI Appendix*, Fig. S2*D*), indicating that the reduced RUSH-IGF2-HA protein levels in cell lysates after biotin treatment are mainly due to the lysosomal degradation.

### TMED10 Is Important for Packaging IGF2 into COPII Vesicles.

**Fig. 2.**
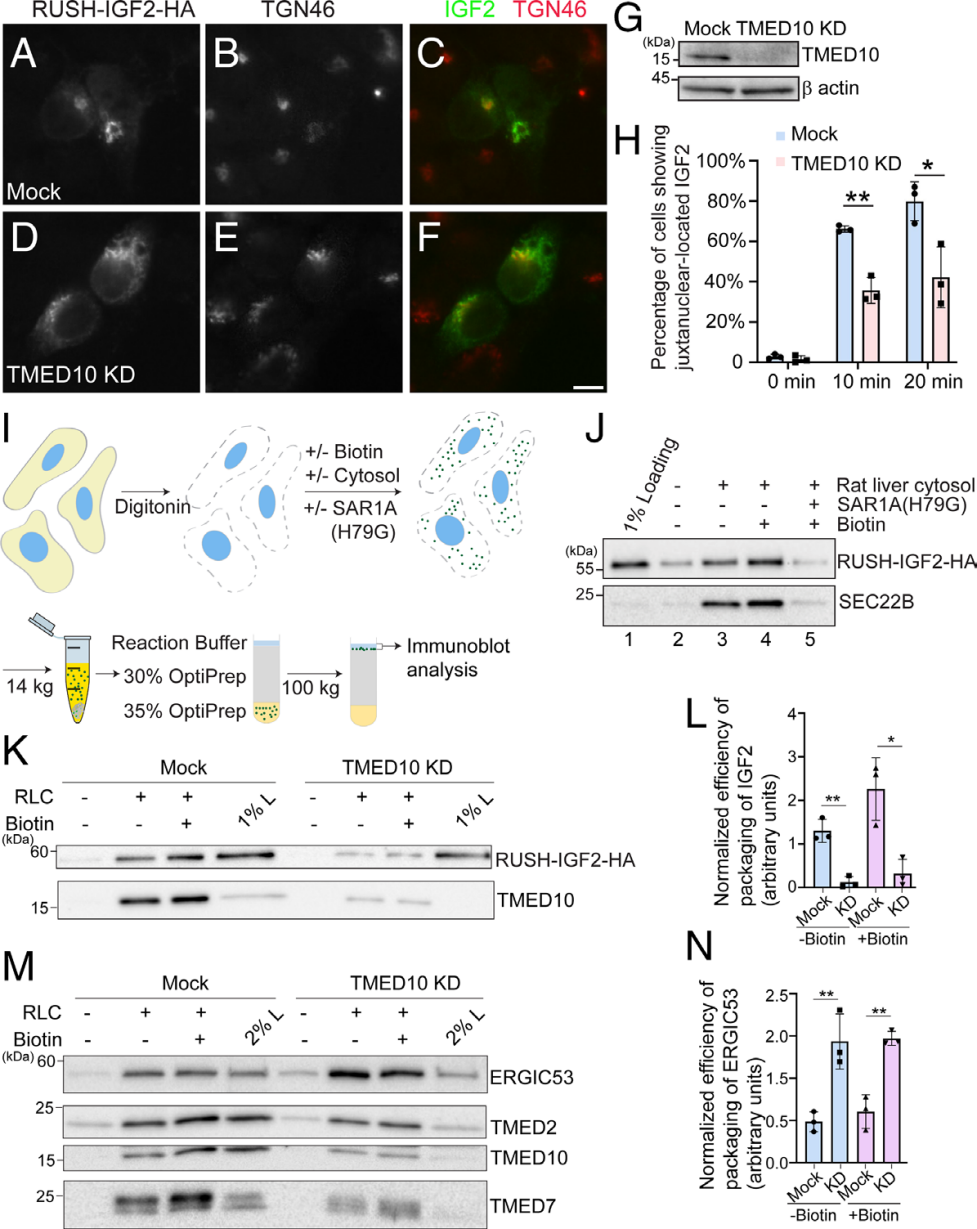
TMED10 mediates the release of IGF2 into COPII vesicles. (*A*–*F*) HeLa cells were transfected with control siRNA or siRNA against TMED10. Day 1 after transfection, cells were retransfected with plasmids encoding Str-KDEL and RUSH-IGF2-HA. On day 3 after knockdown, cells were preincubated with cycloheximide for 2 h. Then cells were incubated with biotin and cycloheximide for 20 min and the localization of IGF2 was analyzed (Size bar, 10 μm). (*G*) Concurrently, the abundance of TMED10 and β actin in cell lysates before biotin treatment was analyzed by immunoblot. A representative example of three biological repeats was shown in this panel. (*H*) Quantification of the percentage of cells showing juxtanuclear-localized RUSH-IGF2-HA at the indicated time point after biotin treatment (mean ± SD; *n* = 3; >100 cells counted in each experiment). (*I*) A diagram demonstrating the vesicle formation assay. (*J*, *K*, and *M*) The vesicle formation assay was performed using HEK293T cells (*J*) or HEK293T cells transfected with control siRNA or siRNA against TMED10 (*K* and *M*). Vesicle fractions were analyzed by immunoblot. (*L* and *N*) Quantification of the budding efficiency of the indicated proteins from the vesicle formation assay (mean ± SD; *n* = 3). The budding efficiency was quantified by calculating the abundance of RUSH-IGF2-HA in the vesicle fraction normalized to the level of the cargo protein in 1% loading. The value was then normalized to the average value of all experimental groups in each replicated experiment. *P < 0.05; **P < 0.01; ***P < 0.001.

Next, we analyzed which step TMED10 is involved in the secretion of IGF2. We found that knockdown of TMED10 caused a defect in ER-to-Golgi trafficking of RUSH-IGF2-HA (Fig. 2 *A*–*G* and quantification in Fig. 2*H*). The defect was also observed in TMED10 KO cells (*SI Appendix*, Fig. S3 *A*–*R* and quantification in *SI Appendix*, Fig. S3*S*). This defect was rescued by the expression of TMED10-FLAG in TMED10 KO cells (*SI Appendix*, Fig. S3 *T–AB* and quantification in *SI Appendix*, Fig. S3*AC*), suggesting that TMED10 is important for ER-to-Golgi trafficking of RUSH-IGF2-HA. When TMED10-FLAG was coexpressed with RUSH-IGF2-HA in the absence of biotin, it was located at the ER in the majority of the TMED10- and IGF2-coexpressing cells (*SI Appendix*, Fig. S4 *A*–*C*). Under this condition, only around 4% of the coexpressing cells showed detectable localizations of TMED10-FLAG at the juxtanuclear area (*SI Appendix*, Fig. S4*G*). After biotin treatment for 10 min, RUSH-IGF2-HA was located at the juxta-nuclear Golgi area (*SI Appendix*, Fig. S4 *D*–*F*). Under this condition, TMED10-FLAG was colocalized with RUSH-IGF2-HA at the juxta-nuclear Golgi area in ∼60% of the coexpressing cells (*SI Appendix*, Fig. S4 *D*–*F* and quantification in *SI Appendix*, Fig. S4*G*). The percentage of the coexpressing cells showing juxta-nuclear TMED10-FLAG was significantly higher in the presence of biotin than that detected in the absence of biotin (*SI Appendix*, Fig. S4*G*). These analyses indicate that ER retention of IGF2 causes accumulations of TEMD10 at the ER. These analyses also indicate that TMED10 traffics together with RUSH-IGF2-HA from the ER to the Golgi. We then performed a similar analysis in cells coexpressing the RUSH construct of ShhN (RUSH-ShhN-HA) and TMED10-FLAG (*SI Appendix*, Fig. S4 *H*–*N*). Our findings revealed that TMED10-FLAG did not co-traffic with RUSH-ShhN-HA from the ER to the Golgi following biotin treatment (*SI Appendix*, Fig. S4*N*). These analyses indicate that TMED10 is cotransported with IGF2 but not ShhN from the ER to the Golgi.

We hypothesize that TMED10 functions as a cargo receptor to regulate packaging IGF2 into COPII vesicles. To test this hypothesis, we reconstituted the release of IGF2 into COPII vesicles using the vesicle formation assay (24, 26). HEK293T cells transfected with RUSH-IGF2-HA were permeabilized by digitonin. After permeabilization, the semi-intact cells were washed with cold KOAc buffer to remove cytosolic proteins. The semi-intact cells were then incubated at 32 °C with GTP and an ATP regeneration system (ATPrS) in the presence or absence of biotin, rat liver cytosol (RLC) and a GTPase defective mutant of SAR1A, SAR1A (H79G) (Fig. 2*I*). The released vesicles after incubation were then isolated by centrifugation and analyzed by immunoblot (Fig. 2*I*). RUSH-IGF2-HA was detected in the vesicle fraction when the vesicle formation assay was performed in the presence of RLC (Fig. 2*J*, lane 3). We detected biotin-independent budding of RUSH-IGF2-HA suggesting that the RUSH system does not block ER export. The abundance of IGF2 in the vesicle fraction was enhanced when the assay was performed in the presence of biotin likely due to the release from retrieval (Fig. 2*J*, compare lanes 3 and 4). Adding SAR1A (H79G) blocked the vesicular release of RUSH-IGF2-HA (Fig. 2*J*, compare lanes 4 and 5). These results indicate that this assay successfully reconstituted the release of IGF2 into COPII vesicles. Remarkably, knockdown of TMED10 caused a significant reduction in the abundance of RUSH-IGF2-HA in transport vesicles under both conditions (with or without biotin, Fig. 2 *K* and *L*), suggesting that TMED10 is important for packaging RUSH-IGF2 into COPII vesicles.

In yeast, ERV25 (the yeast homolog of human TMED10) forms a heteromeric complex with EMP24, ERP1, and ERP2 (the yeast homologs of human TMED2, 4, and 7, respectively) (27). Their protein levels are interdependent, and these proteins function in a cooperative manner (27). In mammals, TMED10 exists in a hetero-oligomeric complex with TMED2, TMED7, and TMED9 (28, 29). We then analyzed the efficiency of budding of two p24 family proteins, TMED2 and TMED7, and another cargo receptor, ERGIC53, in control cells and in cells knockdown of TMED10. Consistent with previous reports, the abundances of TMED7 and TMED2 in cell lysates were markedly reduced in TMED10 KD cells (Fig. 2*M*), indicating interdependence among TMED family proteins for their stability. Consequently, the abundance of these two cargo proteins in the vesicle fraction was also reduced (Fig. 2*M*). Interestingly, the packaging efficiency of ERGIC53 was found to be enhanced in TMED10 KD cells (Fig. 2 *M* and *N*).

We next analyzed the colocalization between IGF2 and TMED10 utilizing a digitonin-permeabilized cell assay. We have previously demonstrated that this assay locks the ER export process at the sorting step (21), providing a convenient way to analyze the colocalization between cargo receptors and cargo molecules. Cells coexpressing RUSH-IGF2-HA and TMED10-FLAG were permeabilized by digitonin and washed with high salt buffer to remove the endogenous cytosolic proteins. Then cells were incubated with rat liver cytosol, biotin, and GTPγS for 15 min. After incubation, RUSH-IGF2-HA and TMED10-FLAG showed punctate localization patterns (*SI Appendix*, Fig. S5 *A*–*C*). Many IGF2 punctate structures overlapped with the punctate structures of TMED10 (*SI Appendix*, Fig. S5 *A*–*C*, magnified views in *SI Appendix*, Fig. S5 *D*–*I*). This analysis indicates that IGF2 colocalizes with TMED10 upon exiting the ER.

### Residues 112-140 in IGF2 Are Important for ER-to-Golgi Trafficking of IGF2.

**Fig. 3.**
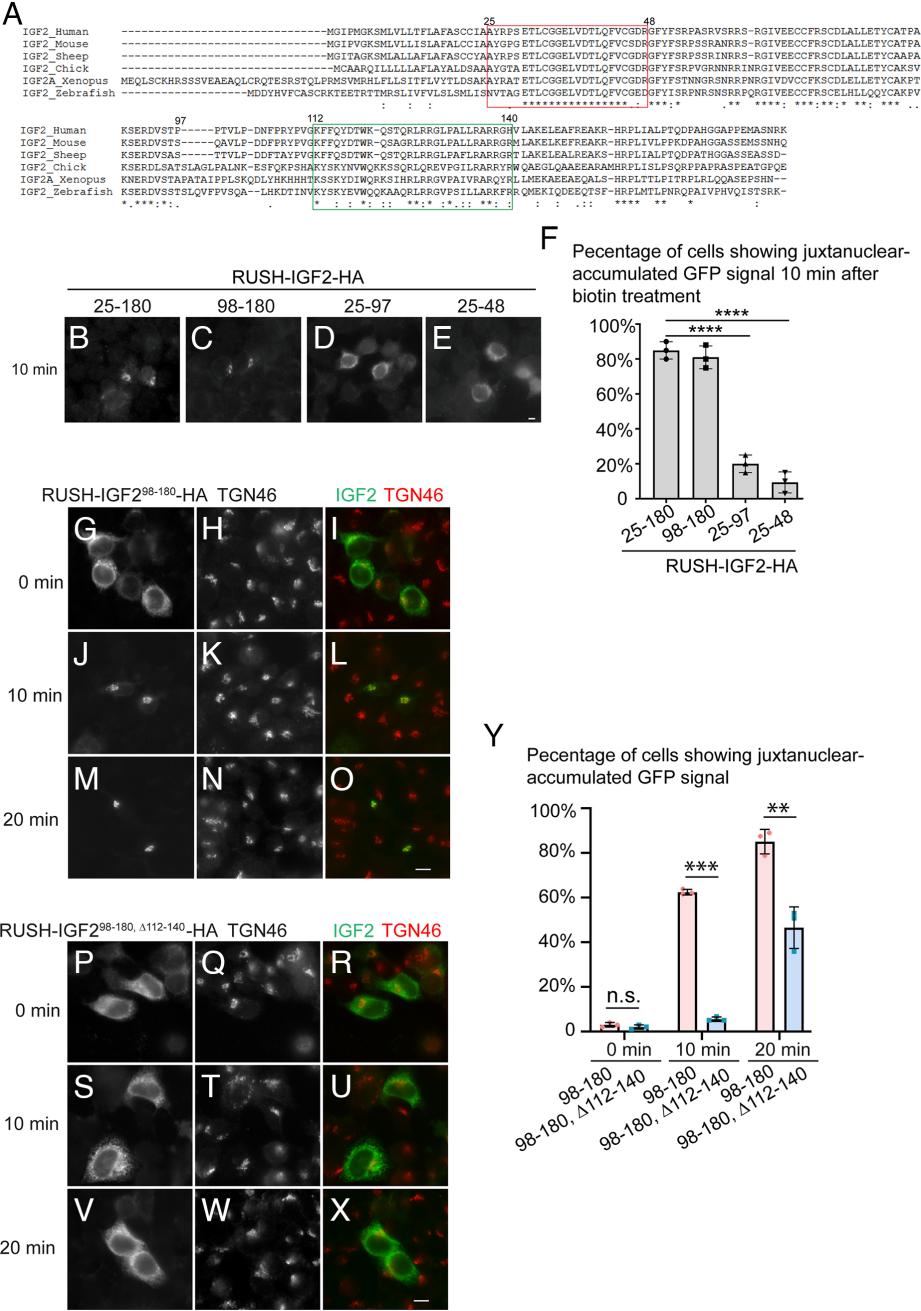
The residues 112-140 in IGF2 are required for ER-to-Golgi trafficking of IGF2. (*A*) Sequence alignment of IGF2 from different species. (*B*–*E* and *G*–*X*) HeLa cells transfected with plasmids encoding the indicated RUSH constructs were incubated with biotin and cycloheximide for the indicated period of time. The localizations of the indicated proteins were analyzed by immunofluorescence (Size bar, 10 μm). (*F* and *Y*) The percentage of cells showing juxtanuclear localization patterns of the RUSH construct after biotin treatment was quantified (mean ± SD; *n* = 3; >100 cells counted in each experiment). **P < 0.01; ***P < 0.001; ****P < 0.0001; n.s., not significant.

The next question we want to address is which motif of IGF2 is the major determinant for IGF2 trafficking and secretion. We performed sequence alignment of different IGF2 orthologues and synthesized different IGF2 truncated proteins; each contains one or several distinct highly conserved regions (Fig. 3*A*). Utilizing the RUSH assay, we tested ER-to-Golgi trafficking of these mutant constructs. Interestingly, we found that RUSH-IGF2^25-48^-HA was located at the ER in over 90% of cells after 10 min biotin treatment (Fig. 3 *E* and *F*). RUSH-IGF2^25-97^- HA also showed a defect of ER-to-Golgi trafficking (Fig. 3 *D* and *F*). In contrast, RUSH-IGF2^98-180^-HA localized at the Golgi apparatus in around 80% of the cells after biotin treatment for 10 min (Fig. 3 *C* and *F*), suggesting that IGF2^98-180^ is the critical part of IGF2 trafficking from the ER to the Golgi.

Sequence alignment indicates that the residues between positions 112 and 140 of IGF2 are conserved across species (Fig. 3*A*, highlighted in the green box). To test whether these residues are important for ER export of IGF2, we generated a RUSH construct of IGF2^98-180^ fragment depleted these residues (RUSH-IGF2^98-180, Δ112-140^-HA). Upon biotin treatment for 10 min or 20 min, RUSH-IGF2^98-180^-HA showed juxtanuclear localization in the majority of cells (Fig. 3 *G*–*O* and *Y*). In contrast, the majority of cells expressing RUSH-IGF2^98-180, Δ112-140^-HA showed an ER pattern (Fig. 3 *P*–*Y*). Further analyses indicate that residues 112 to 140 in IGF2 are sufficient for SBP-EGFP to be delivered from the ER to the Golgi with an efficiency that is similar to full-length IGF2 (*SI Appendix*, Fig. S6 *A*–*M*). In summary, these observations provide evidence demonstrating that IGF2^112-140^ is the ER-to-Golgi transport motif of IGF2.

### IGF2 Interacts with the GOLD Domain of TMED10, and This Interaction Depends on Residues 112-140 in IGF2.

**Fig. 4.**
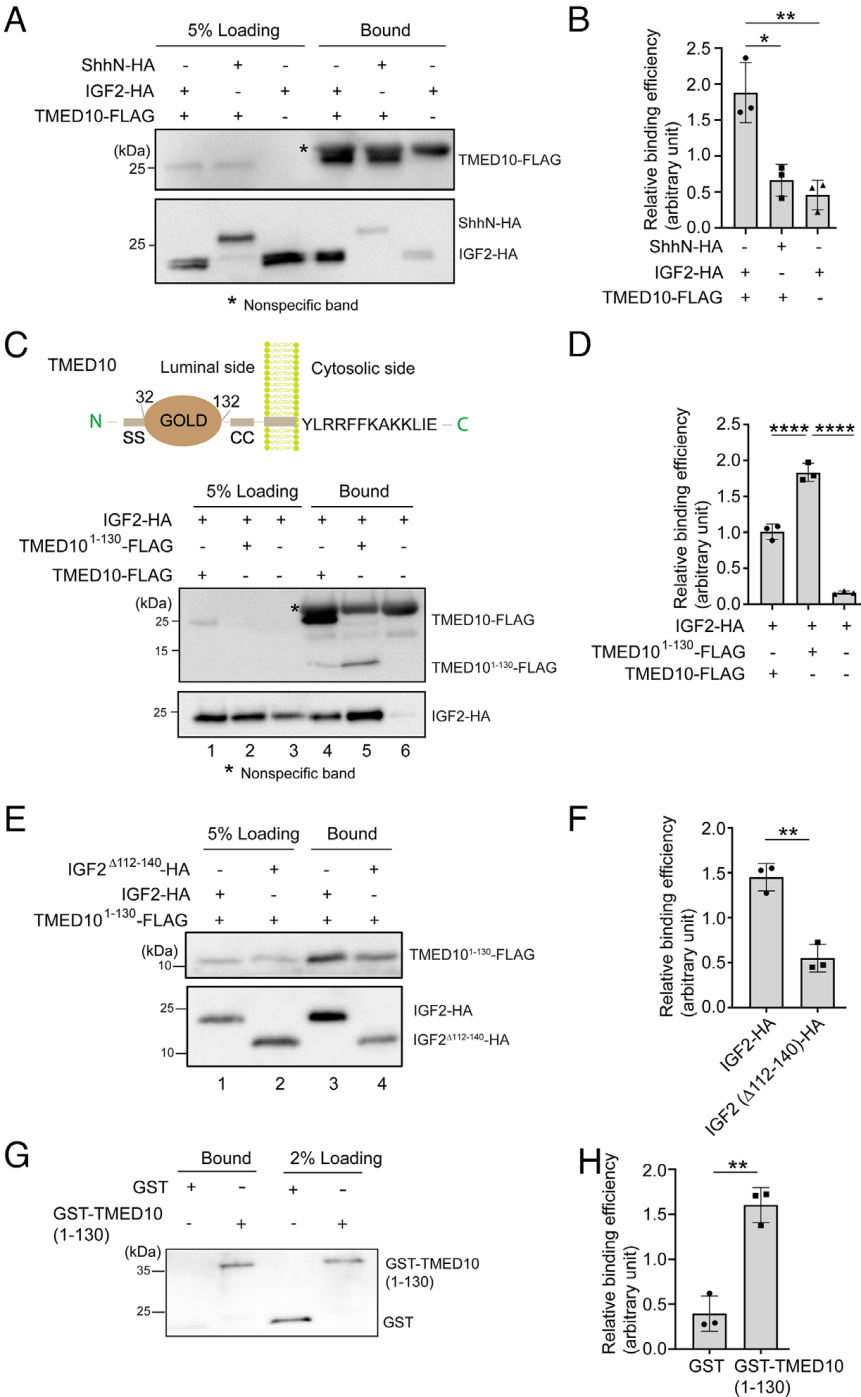
Residues 112-140 in IGF2 directly interact with the GOLD domain of TMED10. (*A*, *C*, and *E*) HEK293T cells were cotransfected with plasmids encoding the indicated constructs. Day 1 after transfection, the cells were treated with 2 mM DSP, and the cell lysates were incubated with beads conjugated with anti-FLAG antibodies. The bound proteins were analyzed by immunoblot. (*B*, *D*, and *F*) Quantification of relative levels of indicated proteins that coimmunoprecipitated with the FLAG-tagged proteins (mean ± SD; *n* = 3). The quantification was performed by calculating the abundance of the bound protein normalized to the abundance of the protein in the loading. The value was then normalized to the average value of all of the experimental groups in each biological repeat. (*G*) Peptides corresponding to the 112-140 residues in IGF2 were covalently linked to thiopyridone sepharose 6B, incubated with purified GST or GST-tagged human TMED10 GOLD domain (residues 1-130). After incubation, the bound proteins were analyzed by immunoblot. (*H*) Levels of GST-TMED10^1-130^ bound to the IGF2 peptides were quantified (mean ± SD; *n* = 3). The quantification is normalized to the average level of GST and GST-TMED10 (1-130) that bound to the IGF2 peptides in each biological repeat. *P < 0.05; **P < 0.01; ****P < 0.0001.

We then performed co-IP experiments using HEK293T cells cotransfected with plasmids encoding FLAG-tagged TMED10 (TMED10-FLAG) and HA-tagged IGF2 or ShhN (IGF2-HA or ShhN-HA). A cross-linker DSP was used in the co-IP experiments to stabilize the interaction. The co-IP assay revealed that IGF2-HA bound TMED10-FLAG in cell lysates (Fig. 4*A*). The percentage of IGF2-HA in cell lysates that bound to TMED10-FLAG was significantly higher than the percentage of ShhN-HA in cell lysates that bound to TMED10-FLAG (Fig. 4 *A* and *B*), indicating TMED10 specifically interacts with IGF2.

TMED family proteins have highly conserved structures. The luminal part of TMED proteins consists of the signal sequence (SS), the Golgi dynamics (GOLD) domain, and the coiled-coil (CC) region (30, 31) (Fig. 4*C*). The cytosolic portion of many TMED proteins contains two C-terminal hydrophobic residues that promote ER export (32) and dilysine motifs (KK) that are important for ER retrieval (33). TMED10, while it possesses a dilysine motif within its cytosolic domain, lacks hydrophobic residues at its C terminus. As TMED10 forms a complex with other proteins from the TMED10 family, TMED10 might be enriched into COPII vesicles through the ER export motif found in other members of the TMED family. The GOLD domain is implicated in recognizing cargo proteins (34). We then generated FLAG-tagged TMED10^1-130^, which contains the SS motif and the GOLD domain, to test whether the GOLD domain is sufficient for the interaction. Strikingly, the abundance of IGF2-HA that bound to TMED10^1-130^-FLAG was significantly higher than that bound to the full-length TMED10-FLAG (Fig. 4*C*, compare lanes 4 and 5, and Fig. 4*D*), suggesting that TMED10 binds IGF2 through its luminal GOLD domain.

Since the ER-to-Golgi transport of IGF2 depends on its residues between 112 and 140, we next tested whether this motif is important for the IGF2-TMED10 interaction. We found that depleting this motif significantly reduced the abundance of IGF2-HA that bound to TMED10-FLAG (Fig. 4*E*, compare lanes 3 and 4, and Fig. 4*F*). We then performed a peptide binding assay to study whether this interaction is direct. Synthesized peptides corresponding to the 112-140 residues of IGF2 (IGF2^112-140^) were covalently linked to beads. The beads were then incubated with purified GST or GST-tagged TMED10 GOLD domain (GST-TMED10^1-130^). The result shows that the abundance of GST-TMED10^1-130^ that bound to IGF2^112-140^ was significantly higher than the abundance of GST that bound to the peptides (Fig. 4 *G* and *H*), suggesting that the ER export motif of IGF2 interacts with the GOLD domain of TMED10 directly.

### TMED10 Is Important for the Secretion of IGF2 from C2C12 Cells for Muscle Stem Cell Differentiation.

We then tested whether TMED10 is important for the secretion of IGF2 from mouse C2C12 myoblasts. We collected the medium incubated with C2C12 cells transfected with control siRNA or siRNA against TMED10. The proteins in the medium were TCA precipitated and then analyzed by label-free quantitative mass spectrometry to compare the abundances of proteins detected in the medium from the two experimental groups (Dataset S1, sheet 1). The abundance of IGF2 was at least 1.9-fold higher in the medium of control cells compared to the medium of TMED10 knockdown cells in each of the two replicated experiments (Dataset S1, sheet 3, highlighted in Red). Along with IGF2, we identified 283 proteins that also exhibited a similar increase in abundance in the medium of control cells (Dataset S1, sheet 2). Of these proteins, 53 are secretory proteins (Dataset S1, sheet 3), while the others are transmembrane, GPI-anchored, cytoplasmic, peripheral membrane, or nuclear proteins. We suspect that those nonsecretory proteins including cytoplasmic, peripheral membrane, transmembrane, GPI-anchored, and nuclear proteins might originate from dying cells during the cell culturing process or from extracellular vesicles secreted by cells. The abundance of these proteins in the medium may be indirectly influenced by the knockdown of TMED10 in C2C12 cells.

**Fig. 5.**
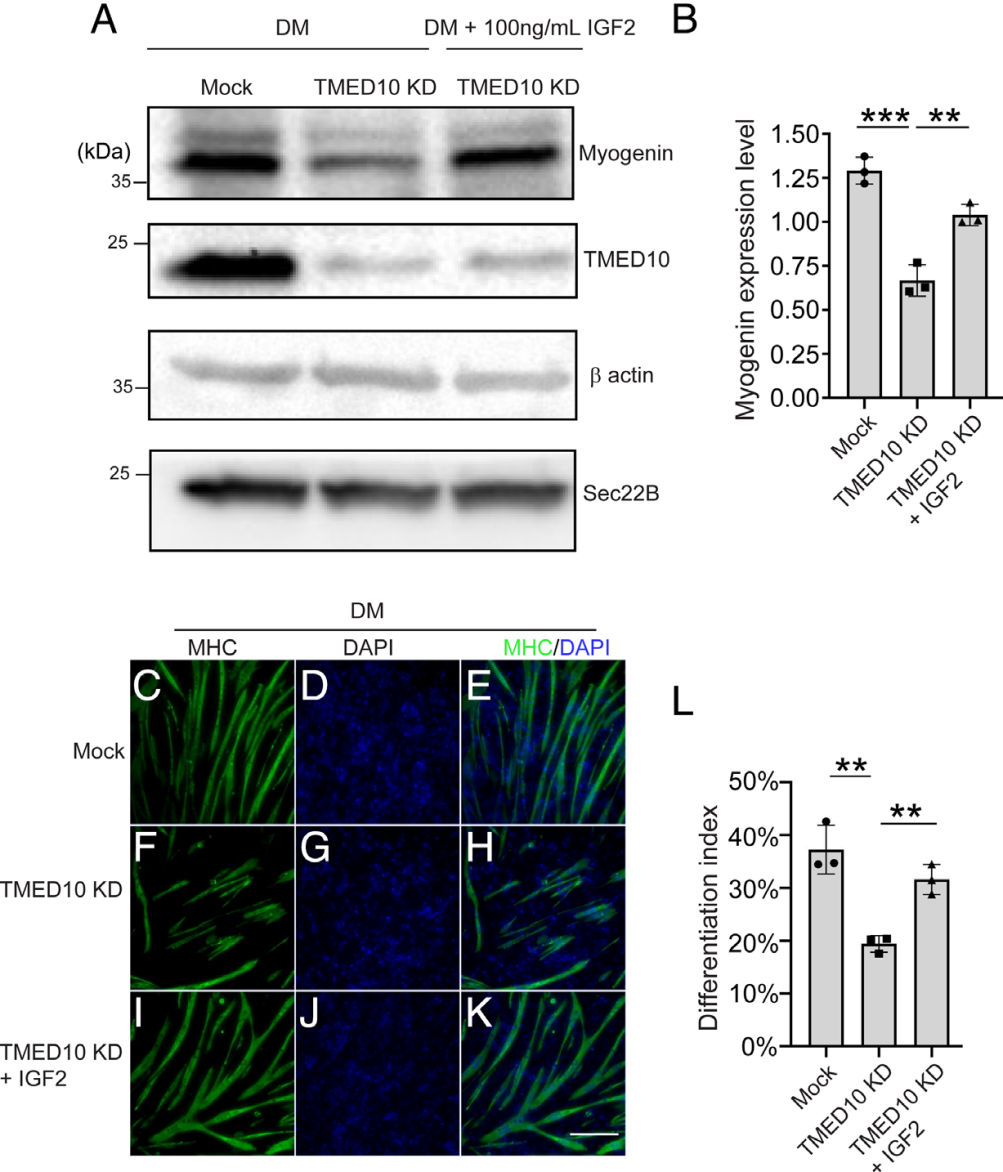
TMED10 plays important roles in the secretion of IGF2 in C2C12 cells for muscle stem cell differentiation. (*A*) C2C12 cells transfected with control siRNA or siRNA against TMED10 were incubated with the differentiation medium (DM) with or without 100 ng/mL purified IGF2. After incubation for 3 d, the expression of the indicated proteins was analyzed by immunoblot. (*B*) Relative levels of the indicated proteins after cell differentiation assay were quantified (mean ± SD; *n* = 3). The abundance of myogenin in each experimental group was normalized to the average abundance of myogenin in all of the three experimental groups in each biological repeat. (*C*–*K*) C2C12 cells transfected with control siRNA or siRNA against TMED10 were incubated with the DM with or without 100 ng/mL purified IGF2 for 3 d. The morphology of the myotube that labeled with MHC was analyzed by immunofluorescence (Size bar, 200 μm). (*L*) Quantification of the differentiation index in each of the indicated experimental groups (mean ± SD; *n* = 3). The differentiation index was quantified by calculating the percentage of the number of nuclei in myosin heavy chain (MHC)-positive cells versus the total number of nuclei after the C2C12 differentiation assay. ***P < 0.001; **P < 0.01.

Expression of the myoblast differentiation marker, myogenin, was significantly reduced in TMED10 knockdown cells incubated with the differentiation medium (DM) when compared to the control cells incubated at the same condition (Fig. 5*A*). Adding purified IGF2 into the differentiation medium rescues the expression of myogenin in TMED10 knockdown cells (Fig. 5*A* and quantification in Fig. 5*B*). These results indicate that TMED10 plays an important role in myoblast differentiation by functioning as a cargo receptor to enrich IGF2 into COPII vesicles, a process that is crucial for the secretion of IGF2. To further analyze the rescue effects, we analyzed the myotube formation of C2C12 cells by staining the myosin heavy chain (MHC), a marker protein of the myotubes. Consistent with our western blot result, the myotube became much shorter and thinner after knockdown of TMED10 (Fig. 5 *F*–*H*). After incubation with purified IGF2, myotube formation was rescued, and myotubes became longer and thicker (Fig. 5 *I*–*K*). We then quantified the differentiation index after the C2C12 differentiation assay. The differentiation index was significantly reduced in TMED10 KD cells and this defect was recused by purified IGF2 (Fig. 5*L*). These analyses indicate that TMED10 regulates C2C12 differentiation through an autocrine manner. Taken together, we revealed that TMED10 is important for packaging IGF2 into COPII vesicles to deliver IGF2 from the ER to the Golgi, and this step is critical for myoblast differentiation.

### Sortilin Is Another Cargo Client of TMED10.

**Fig. 6.**
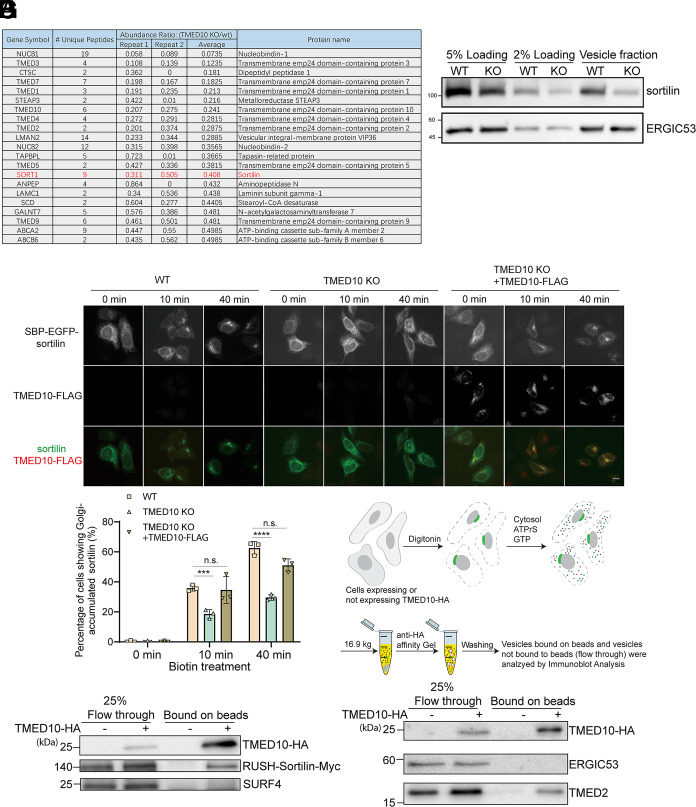
TMED10 regulates ER-to-Golgi transport of sortilin. (*A*) Table showing the list of transmembrane proteins that are less represented in the vesicle fraction in the TMED10 KO group compared to the WT group (average fold change < 0.5). (*B*) The vesicle formation assay was performed using HeLa WT or HeLa TMED10 KO cells. The indicated proteins were analyzed by immunoblot. (*C*) WT or TMED10 KO HeLa cells were transfected with SBP-EGFP-sortilin and Str-KDEL in the presence or absence of TMED10-FLAG. Twenty-four hours after transfection, cells were preincubated with cycloheximide for 2 h. Then, cells were incubated biotin with cycloheximide for the indicated time points. The localizations of the indicated proteins were then analyzed by immunofluorescence (Size bar, 10 μm). (*D*) Quantification of the percentage of cells showing juxta-nuclear-located sortilin (mean ± SD; *n* = 3; >100 cells counted for each experiment). ***P < 0.001; ****P < 0.0001; n.s., not significant. (*E*) A diagram demonstrating the vesicle immunoprecipitation assay. (*F* and *G*) The vesicle formation assay was performed in untransfected HeLa cells or in cells co-transfected with plasmids encoding TMED10-HA and plasmids encoding RUSH-sortilin-Myc. Subsequently, vesicles enriched with TMED10-HA were immunoisolated, and the abundance of the indicated proteins in the immunoisolated vesicles (bound on beads) and the abundance of the indicated proteins in the vesicles that were not immunoisolated (flow through) were analyzed by immunoblot. Data shown in panels *F* and *G* are representative example of three biological repeats.

Next, we sought to identify other cargo proteins that depends on TMED10 to be enriched into transport vesicles. We have previously developed a vesicle formation assay in combination with a label-free quantitative mass spectrometry approach and this approach revealed the cargo clients of two ER cargo receptors, ERGIC53 and SURF4 (26). Here, we utilized a similar approach to uncover the cargo clients of TMED10. A large-scale vesicle formation assays were performed using donor membranes provided by wild-type (WT) or TMED10 KO HeLa cells. A label-free quantitative mass spectrometry analysis was then conducted to compare the protein profiling of vesicles produced from these two experimental groups (the WT group and the TMED10 KO group, Dataset S2). We detected peptides that match TMED10 in the vesicle fraction generated by the TMED10 KO cells. A possible explanation is that a negligible quantity of TMED10 may persist in rat liver cytosol prepared from rat livers, which may associate with vesicles after the vesicle formation assay. Although these residual amounts of proteins are not detectable through immunoblotting, they may be detected using mass spectrometry that has the capacity to detect proteins in the low picogram range. We found that the abundance of a series of transmembrane proteins is greatly reduced in the vesicle fraction in the TMED10 KO group based on two biological repeats (Fig. 6*A*, average fold change of TMED10 KO/WT < 0.5). These identified transmembrane proteins including several p24 family proteins: TMED1, TMED2, TMED3, TMED4, TMED5, TMED7, and TMED9 (Fig. 6*A*). This decrease is presumably caused by the degradation of these TMED proteins induced by the depletion of TMED10, thereby reducing their presence not only within the cells but also in the vesicle fraction.

In addition, we identified a Golgi- and plasma-membrane localized transmembrane protein, sortilin, that depends on TMED10 to be enriched into transport vesicles (Fig. 6*A*, highlighted in red, Fig. 6*B*). The total level of sortilin was also greatly decreased in both cell lysates and vesicle fraction of the KO group (Fig. 6*B*). To investigate whether TMED10 is important for the ER-to-Golgi trafficking of sortilin, we generated a RUSH construct of sortilin and performed the RUSH transport assay in WT or TMED10 KO HeLa cells. We found that the ER-to-Golgi transport of sortilin was strongly impaired in KO cells (Fig. 6 *C* and *D*): after biotin treatment for 40 min, the majority of sortilin was trapped at the ER. This defect was rescued by transfecting TMED10-FLAG in KO cells (Fig. 6 *C* and *D*). These results indicate that TMED10 is also essential for ER-to-Golgi trafficking of sortilin. We then performed the vesicle formation assay and immunoisolated vesicles enriched with TMED10-HA (Fig. 6*E*). Our findings revealed that these isolated vesicles contained RUSH-sortilin-Myc and TMED2 (Fig. 6 *F* and *G*), but not SURF4 and ERGIC53 (Fig. 6 *F* and *G*). This analysis indicates that sortilin and TMED2 reside in the same vesicles as TMED10, unlike SURF4 and ERGIC53.

### Sortilin Regulates TGN Export of IGF2.

**Fig. 7.**
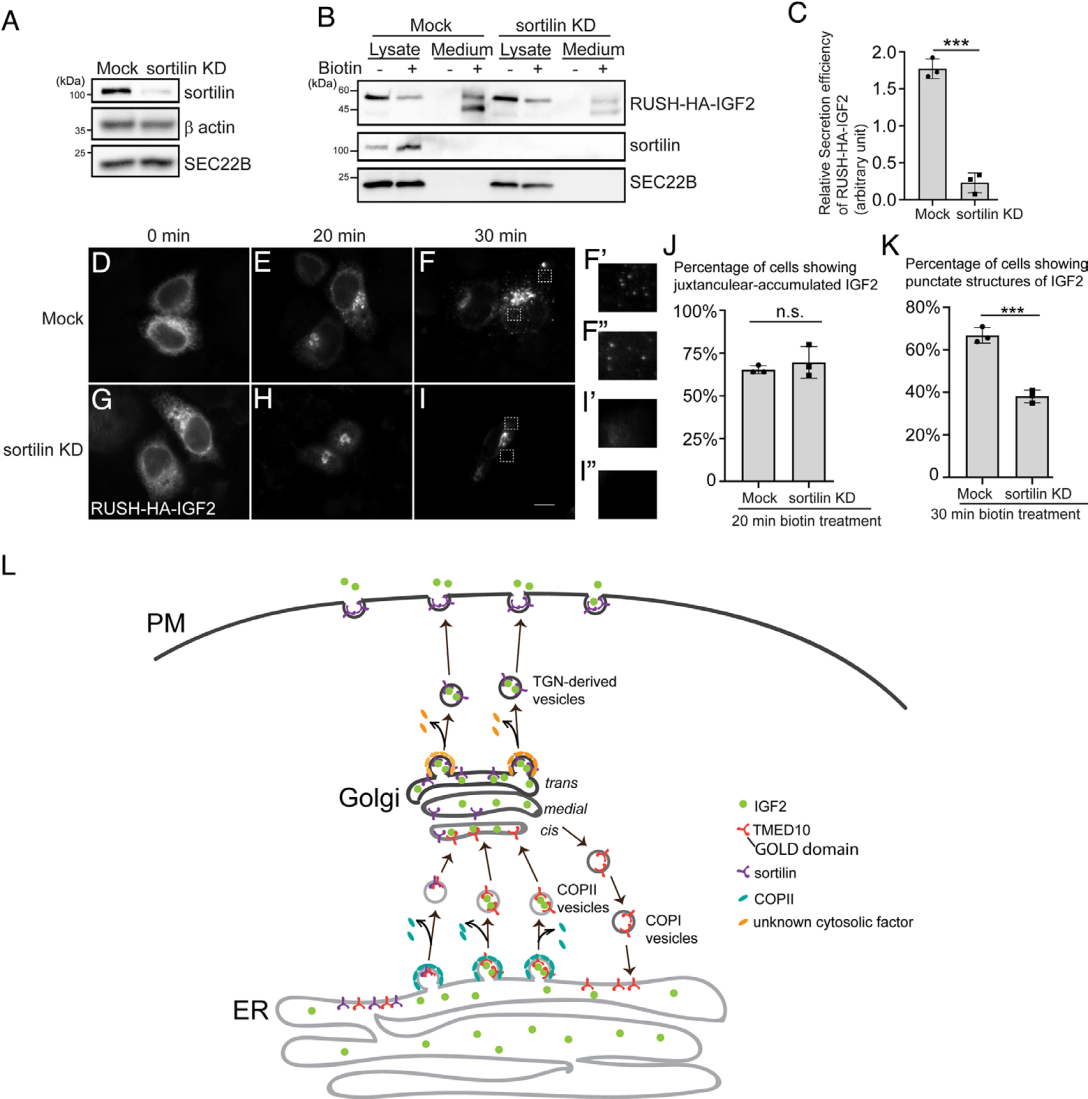
Sortilin regulates TGN export of IGF2. (*A*) HeLa cells were transfected with control siRNA or siRNA against sortilin. Day 2 after transfection, the level of the indicated proteins in cell lysates was analyzed by immunoblot. (*B*) HeLa cells were transfected with control siRNA or siRNA against sortilin. Day 1 after transfection, cells were retransfected with plasmids encoding Str-KDEL and RUSH-HA-IGF2. On day 3 after knockdown, cells were preincubated with cycloheximide for 2 h. Then, cells were incubated with biotin and cycloheximide for 2 h. After biotin incubation, the level of RUSH-HA-IGF2 in the medium and in cell lysates was analyzed by immunoblot. (*C*) Quantification of the abundance of secreted IGF2 normalized to the abundance detected in the cell lysate group (mean ± SD; *n* = 3). The value in each experimental group was normalized to the average value in the Mock group and the sortilin KD group in each biological repeat. (*D*–*I*″) HeLa cells were transfected with control siRNA or siRNA against sortilin. Day 1 after transfection, cells were retransfected with plasmids encoding Str-KDEL and RUSH-HA-IGF2. On day 3 after knockdown, cells were preincubated with cycloheximide for 2 h. Then, cells were incubated with cycloheximide and biotin for the indicated time, and the localization of RUSH-HA-IGF2 was analyzed (Size bar, 10 μm). The magnified view of the indicated area in panels *F* and *I* is shown in panels F′, F″, I′, and I″. (*J*) Quantification of the percentage of cells showing juxtanuclear-located RUSH-HA-IGF2 (mean ± SD; *n* = 3; >100 cells counted in each experimental group). (*K*) Quantification of the percentage of cells showing punctate patterns of RUSH-HA-IGF2 (mean ± SD; *n* = 3; >100 cells counted in each experimental group). (*L*) The proposed model demonstrating the dual functions of TMED10 in mediating IGF2 trafficking along the secretory pathway: 1) the GOLD domain of TMED10 recognizes the 112-140 residues of IGF2 to enrich IGF2 into COPII vesicles; 2) TMED10 also regulates ER export of sortilin, which is important for TGN-to-plasma membrane trafficking of IGF2. ***P < 0.001; n.s., not significant.

Our previous analyses indicate that ER-to-Golgi trafficking of IGF2 and sortilin is mediated by TMED10. Sortilin mediates insulin-dependent glucose transport in myocytes and is crucial for myogenesis (16, 35). Interestingly, we found that knockdown of sortilin significantly reduced the efficiency of RUSH-HA-IGF2 secretion after biotin treatment (Fig. 7 *A*–*C*), indicating that sortilin plays a crucial role in IGF2 secretion. To study whether sortilin is required for ER-to-Golgi trafficking or TGN-to-plasma membrane transport of IGF2, we analyzed the RUSH-HA-IGF2 trafficking in sortilin KD HeLa cells at different time points after biotin treatment. The percentage of cells showing juxta-nuclear localized RUSH-HA-IGF2 was similar in Mock and sortilin KD group after biotin treatment for 20 min (Fig. 7 *D*, *E*, *G*, *H*, and *J*), suggesting that knockdown of sortilin did not affect the ER-to-Golgi trafficking of RUSH-HA-IGF2. RUSH-HA-IFG2 showed punctate structures in the cell periphery in over 60% of cells after biotin treatment for 30 min. We hypothesize that these punctate structures are TGN-derived vesicles enriched with RUSH-HA-IGF2. The percentage of cells showing punctate structures of RUSH-HA-IGF2 was significantly decreased in sortilin KD group compared to the control group (Fig. 7 *F*, *I*, and *K*). These analyses demonstrated that sortilin is important for TGN export but not ER export of IGF2.

## Discussion

Secretion of soluble signaling proteins from the producing cells is tightly related to the downstream signaling pathway in targeted cells. Although a series of factors are identified to regulate the expression of IGF2 such as mTOR, PLD1, and miR-125b (4, 36–38), the cargo receptors that regulate the biosynthetic trafficking of IGF2 remain largely unknown. Here, we found that two transmembrane proteins, TMED10 and sortilin, cooperatively mediate IGF2 secretion along the secretory pathway. Based on our study, we propose that the secretion of IGF2 is achieved by several steps (Fig. 7*L*). First, the correctly folded ER-localized pro-IGF2 is captured into COPII vesicles by the direct interaction between the IGF2^112-140^ motif and TMED10 GOLD domain. Second, vesicles containing pro-IGF2 and TMED10 are delivered to the Golgi apparatus where the pro-IGF2 receives O-glycosylation modifications and cleavage. At the Golgi, TMED10 is disassociated from IGF2 and is recycled to the ER by COPI vesicles. In addition, TMED10 also mediates ER export of sortilin. After reaching TGN, sortilin regulates TGN-to-cell surface delivery of IGF2 (Fig. 7*L*).

TMED10 is a member of the p24 family. A null mutation in TMED10 results in early embryonic lethality in mice (39). The inactivation of one allele of TMED10 in mice causes dilation of Golgi cisternae (39) and knockdown of TMED9 in HeLa cells caused dispersal of the Golgi (40). TMED10 negatively regulates autophagy, and the expression of TMED10 is reduced in Alzheimer’s disease patients (41). The yeast homologue of TMED10 forms a complex with the yeast homologue of TMED2 and this complex regulates ER-to-Golgi transport of a GPI-anchored protein, Gas1p (42). In mammalian cells, TMED10 is also shown to regulate surface delivery of a GPI-anchored proteins (43). GTP-bound form of Rab21 was shown to interact with TMED10 and regulate localizations of TMED10 at the Golgi (44). Immunoprecipitation results revealed that both TMED10 and TMED2 showed a preference to interact with Sec24C and Sec24D, indicating that Sec24C and Sec24D are two Sec24 isoforms involved in TMED10 mediated protein trafficking at the ER (45). The majority of p24 family proteins are primarily located within the luminal side of organelle membranes. Their asymmetric nature imposes a curvature that is opposite to the curvature needed for vesicle budding, thereby changing the physical characteristics of membranes (46). It has been shown that the scaffolding function of the cargo adaptor Lst1p, the yeast homologue of Sec24, and the outer COPII coat Sec13p are essential to counter the resistance caused by the p24 proteins and facilitate vesicle formation at the ER (46, 47).

Upon reaching the Golgi, cargo molecules dissociate from their clients. We have previously demonstrated that proteoglycans compete with SURF4 to interact with Shh at the Golgi, thereby causing SURF4 to be dissociated from its client (2). P24 family proteins have been shown to interact with the remodeled GPI-APs to enrich them into COPII vesicles (48, 49). This interaction is pH-dependent, suggesting that they may be dissociated from each other at the Golgi due to pH changes (48). Moreover, p24 proteins have been demonstrated to retrieve escaped, unremodeled GPI-anchored proteins from the Golgi, returning them to the ER within COPI vesicles (49). This suggests that p24 proteins play a crucial role in monitoring anchor remodeling to ensure accurate trafficking of GPI-APs (49). The transmembrane domain of TMED2 (but not TMED10) has been found to interact specifically with a sphingomyelin, SM18. This interaction facilitates efficient retrograde COPI-dependent trafficking and is implicated to modulate the equilibrium between monomeric and oligomeric states of TMED2 (50). An interesting future direction is to investigate how TMED10 is dissociated from IGF2 at the Golgi and whether ER retrieval of TMED10 depends on the TMED2-SM18 interaction.

Intriguingly, TMED10 is also shown to function as a protein channel to mediate the unconventional protein secretion (UPS) of a group of leaderless proteins including IL1β, IL-1α, HSPB5, Tau, and Annexin A1 (51). Unlike the TMED10-mediated conventional secretory pathway, TMED10-channeled UPS requires direct or indirect interactions between UPS cargoes and TMED10 C-terminal tail (31). In the UPS pathway, TMED10, triggered by the production of UPS cargoes, is found to form a higher-order mono-oligomer which stabilizes the TMED10 protein channel on the ERGIC membranes for UPS protein translocation (31). If the oligomeric form of TMED10 forms such a channel, the pore of this channel would be highly hydrophobic as the transmembrane residues of TMED10 are predominantly hydrophobic. This would create an energy barrier for the passage of IL1β and other unconventional cargo proteins. Thus, it remains to be elucidated how a homo-oligomer of TMED10 can translocate IL1β into the luminal side of membranes. In addition, IL1β and other unconventional secretory proteins are found to be efficiently secreted upon infection-induced permeabilization of the plasma membrane of immune cells (52), suggesting a substantial fraction of these cargo molecules are located in the cytoplasm of immune cells. Further analysis is needed to determine the proportion of IL1β that resides within the cytoplasm versus the luminal side of the ERGIC. It remains unclear whether TMED10 forms mono-oligomer or hetero-oligomer with other TMED proteins to regulate IGF2 trafficking. Deleting the GOLD domain abolished TMED10 mono-oligomerization, indicating that the integrity of the GOLD domain is crucial for the mono-oligomerization of TMED10 (31). The direct interaction between IGF2 and TMED10 GOLD domain may interfere with TMED10 mono-oligomerization. In addition, the quantitative mass spectrometry analysis indicates that TMED1, 2, 3, 7, and 9 showed a large decrease in TMED10 KO vesicles (Fig. 6*A*). Thus, we hypothesized that TMED10 and other p24 family proteins form hetero-oligomers to package IGF2 into COPII vesicles.

In addition to the selective capture mechanism, bulk flow is another approach that exports soluble or membrane-associated proteins from the ER (10). Export by bulk flow does not rely on cargo receptors or export motifs on cargoes. Instead, proteins exported by bulk flow were packaged into COPII vesicles by default. Utilizing the RUSH assay, we found that RUSH-IGF2^98-180^-HA without the 112-140 aa motif showed a kinetic delay in ER-to-Golgi transport (Fig. 3 *P*–*Y*). In contrast, fusing the SBP-EGFP tag with IGF2^112-140^ motif efficiently brings SBP-EGFP to the Golgi (*SI Appendix*, Fig. S6), indicating that bulk flow is not an efficient approach in mediating ER-to-Golgi trafficking of IGF2. Cross-linking and peptide binding experiments revealed a direct interaction between TMED10 GOLD domain and the IGF2^112-140^ motif, suggesting that TMED10 directly mediates the ER export of IGF2.

Sortilin is a single-pass transmembrane protein belonging to the vacuolar protein sorting 10 protein (Vps10p) family (12). Although functional roles of sortilin have been extensively studied, the molecular mechanisms mediating the biosynthetic trafficking of sortilin remain largely unclear. Here, we revealed that TMED10 functions as a cargo receptor that mediates the ER-to-Golgi transport of sortilin. In addition, we demonstrate that sortilin is important for the post-Golgi trafficking of IGF2. These findings suggest that TMED10 also indirectly mediates TGN export of IGF2 by regulating the ER-to-Golgi trafficking of sortilin.

In summary, our study provides insights into the molecular machinery that mediates the trafficking of IGF2 along the secretory pathway to perform its physiological functions. Dysregulation of IGF2 activities is a candidate risk factor for tumorigenesis and is related to multiple disorders, such as Beckwith–Wiedemann syndrome, Silver–Russell syndrome, and Doege–Potter syndrome (53–55). The uncovered cellular factors and protein interactions that are important for the secretion of IGF2 provide therapeutic targets to down-regulate IGF2 signaling by blocking IGF2 secretion.

## Materials and Methods

### Constructs, Reagents, Cell Culture, Immunofluorescence, and Transfection.

Cell lines, cDNAs, siRNAs, antibodies, cell culture, immunofluorescence, and transfection were described in *SI Appendix*.

### Immunoprecipitation, Protein Purification, RUSH Assay, Binding Assay, and Vesicle Formation Assay.

Immunoprecipitation, protein purification, RUSH assay, binding assay, and vesicle formation were performed as described previously (21, 56–58). Vesicle immunoprecipitation assay was performed as described previously (56).

### Muscle Stem Cell Differentiation Assay.

Undifferentiated C2C12 cells were cultured with DMEM containing 20% FBS and 1% penicillin–streptomycin mix. To induce differentiation, C2C12 cells transfected with control siRNA or siRNA against TMED10 were incubated with DMEM containing 2% horse serum and 1% penicillin–streptomycin in the presence or absence of 100 ng/mL purified IGF2 (R&D Systems, Catalog number: 792-MG) for 3 d. Then, the cells were analyzed by immunoblot or immunofluorescence.

### Sample Preparation for Label-Free Quantitative MS Analysis.

This procedure was described in *SI Appendix*.

## Supplementary Material

Appendix 01 (PDF)

Dataset S01 (XLSX)

Dataset S02 (XLSX)

## Data Availability

All study data are included in the article and/or supporting information.
