# Supplementary material for: TMED10 mediates the trafficking of insulin-like growth factor 2 along the secretory pathway for myoblast differentiation
Source: Proc Natl Acad Sci U S A. 2023 Nov 6;120(46):e2215285120. doi: 10.1073/pnas.2215285120 (PMC10655563; doi:10.1073/pnas.2215285120)
Supplement: Supplementary file 1 — Appendix 01 (PDF) [file pnas.2215285120.sapp.pdf]

Supplemental Information (SI) Appendix

TMED10 mediates the trafficking of insulin like growth factor 2 along the secretory pathway for myoblast differentiation

Tiantian Li [a,1], Feng Yang[a,1], Youshan Heng[a] , Shaopu Zhou[a] , Gang Wang[a], Jianying Wang[b], Jinhui Wang[a], Xianwei Chen[a], Zhong-Ping Yao[b,c,2], Zhenguo Wu[a,2] and Yusong Guo[a,d,e, 2]

a. Division of Life Science and State Key Laboratory of Molecular Neuroscience, The Hong Kong University of Science and Technology, Hong Kong, China;

b. State Key Laboratory of Chemical Biology and Drug Discovery, Research Institute for Future Food, Research Centre for Chinese Medicine Innovation, and Department of Applied Biology and Chemical Technology, The Hong Kong Polytechnic University, Hung Hom, Kowloon, Hong Kong, China;

c. State Key Laboratory of Chinese Medicine and Molecular Pharmacology (Incubation) and Shenzhen Key Laboratory of Food Biological Safety Control, Hong Kong Polytechnic University Shenzhen Research Institute, Shenzhen 518057, China;

d. Hong Kong University of Science and Technology Shenzhen Research Institute, Shenzhen 518057, China;

e. Thrust of Bioscience and Biomedical Engineering, Hong Kong University of Science and Technology, Guangzhou 511453, China.

1, These authors contribute equally to this work;

2, To whom correspondence should be addressed: guoyusong@ust.hk; bczgwu@ust.hk; zhongping.yao@polyu.edu.hk.

Includes:

SI Materials and Methods

SI Figures, Figure S1-S6

SI Materials and Methods

Constructs, reagents, cell culture, immunofluorescence, and transfection

The cDNA encoding human IGF2, human TMED10, human sortilin, and the plasmids encoding Str-KDEL_SBP-EGFP-HA-IGF2 were synthesized from BGI (Beijing, China). The plasmids encoding 3xHA-tagged IGF2 (IGF2-HA), GST-tagged IGF2, 3xFLAG-tagged TMED10 (TMED10-FLAG), Str-KDEL_SBP-EGFP-sortilin, Str-KDEL_SBP-EGFP-sortilin-myc, Str-KDEL_SBP-EGFP-IGF2-HA and truncated versions of IGF2 were generated by standard molecular cloning procedures. The plasmids encoding mutated version of IGF2 were generated by QuikChange II site-directed mutagenesis using plasmids encoding IGF2-HA or Str-KDEL_SBP-EGFP-IGF2-HA as templates. The plasmids encoding siRNA-resistant TMED10-FLAG were generated by QuikChange II site-directed mutagenesis using plasmid encoding TMED10-FLAG as template.

siRNAs against TMED10 were purchased from Ribo-bio (Guangzhou, China). The target sequence against human TMED10 is GTGAGGAGATTCACAAGGA. The target sequence against mouse TMED10 is GTCCTGTACTTCAGCATCT. The target sequence against sortilin is GCACAATCTTTACCTCAGA. The commercial antibodies were rabbit anti-HA (Cell Signaling, catalogue number 3724), rabbit anti-TMED10 (Proteintech, catalogue number 15199-1-AP), mouse anti-MHC (DSHB, catalogue number MF 20) and mouse anti-Myogenin (Santa Cruz Biotechnology, catalogue number SC-12732); sheep anti-TGN46 (BIO-RAD, catalogue number AHP500G), mouse anti-GM130 (BD Bioscience, catalogue number 610823); mouse anti-FLAG (Sigma, catalogue number F3165); mouse anti-β actin antibody (Proteintech, catalogue number 60008-1-Ig); goat anti-GST (GE Healthcare, catalogue number 27-4577-01); mouse anti-TMED2 (Santa Cruz Biotechnology, catalogue number SC-376459); mouse anti-Myc (Cell signaling, catalogue number 2276); rabbit anti-sortilin (Proteintech, catalogue number 12369-1-AP). Rabbit anti-Sec22B antibodies and rabbit anti-ERGIC53 antibodies were kindly provided by Prof. Randy Schekman (University of California, Berkeley, CA, USA). Rabbit anti-TMED7 antibodies were kindly provided by Prof. Pingbo Huang (Hong Kong University of Science and Technology, Hong Kong, SAR).

HeLa cells and HEK293T cell lines were kindly provided by the University of California-Berkeley Cell Culture Facility and were confirmed by short tandem repeat profiling. All cell lines were tested negative for Mycoplasma contamination. HeLa and HEK293T cells were cultured in Dulbecco's Modified Eagle Medium (DMEM) containing 10% fetal bovine serum and 1% penicillin streptomycin mix (Invitrogen). C2C12 cells were cultured in Dulbecco's Modified Eagle Medium (DMEM) containing 20% fetal bovine serum and 1% penicillin streptomycin mix (Invitrogen). Transfection of siRNA or DNA constructs into HeLa cells, or HEK293T cells and immunofluorescence were performed as described previously (1). Images were acquired with a Zeiss Axio Observer Z1 microscope system (Carl Zeiss, Germany) equipped with an ORCA Flash 4.0 camera (Hamamatsu, Japan) or Leica SP8 Confocal Microscope (Leica, Germany).

For CRISPR experiments, sgRNA sequences were ligated into AAV9-U6-Dsred plasmids. Clonal cell lines were derived by diluting cell suspensions to a single cell per well and expanding individual wells. Genotyping of clonal cell lines was performed by Sanger sequencing of target site PCR amplicons of genomic DNA isolated by Puregene kit (Qiagen). sgRNA was as follows: TMED10, 5'TAACGGAAAAGGGCCGCGCC' targeting exon 1 of TMED10.

Retention Using Selective Hook (RUSH) assay

The RUSH assays were performed as described (2). Briefly, HeLa cells were transfected with plasmids encoding Str-KDEL and SBP-EGFP-sortilin or SBP-EGFP-sortilin-myc or different version of the RUSH constructs of IGF2. Day 1 after transfection, cells were incubated in complete medium with 100 ng/µl cycloheximide for 2 hr. Then the cells were incubated in complete medium containing 40 µM biotin (Sigma-Aldrich) and 100 ng/µl cycloheximide (Sigma-Aldrich) for the indicated time. Subsequently, cells were fixed by 4% PFA and mounted on glass slides by ProLongTM Gold Antifade Mountant with DAPI (Invitrogen) for microscope analysis.

To analyze the secretion of IGF2, HeLa cells transfected with plasmids encoding Str-KDEL and different version of the RUSH constructs of IGF2 were incubated in complete medium with 100 ng/µl cycloheximide for 2 hr. Then cells were treated with 100 ng/µl cycloheximide and 40 µM biotin in medium without FBS for the indicated time. Then the secreted proteins were precipitated by TCA precipitation. The cells were collected and lysed by HKT buffer (100 mM KCl, 20 mM Hepes, pH 7.2, 0.5% Triton X-100). The secreted proteins and cell lysates were analyzed by immunoblot.

Immunoprecipitation, protein purification, and binding assay

Immunoprecipitation of FLAG-tagged TMED10 or TMED10$^{1\text{-}130}$ was performed by treating HEK293T cells co-transfected with plasmids encoding the indicated proteins in PBS containing 2 mM dithiobis[succinimidylpropionate] (DSP) and 2 mM $CaCl_2$ at room temperature for 30 min, and then quenched with 25 mM Tris-HCl, pH 7.5. The cells were then lysed in lysis buffer (50mM Tris-HCl, 150mM NaCl, 2mM $CaCl_2$, and 0.1% TX-100, supplemented with proteinase inhibitors (Roche), pH7.5). Subsequently, 500 µl of 0.5 mg/ml cell lysates were incubated with 10 µl of compact anti-FLAG agarose affinity beads at 4 °C overnight. M2-FLAG affinity beads was pre-blocked with 1 hr incubation in blocking buffer (50 mM Tris-HCl, 500 mM NaCl, 2 mM $CaCl_2$, 5% BSA, pH 7.5) to reduce nonspecific binding. After incubation, the beads were washed 3 times with 1 ml of blocking buffer (5%BSA) and 2 times with 1 ml of blocking buffer (-BSA), and the bound material was analyzed by immunoblot.

Purification of GST-tagged TMED10$^{1\text{-}130}$ was performed as described previously (1). Peptide binding assay was performed as described previously (3). Synthetic IGF2 112-140 peptide (KFFQYDTWKQSTQRLRRGLPALLRARRGHC) was purchased from GenScript and coupled to thiopyridone-Sepharose 6B beads (Sigma-Aldrich) via the added C-terminal cysteine residue. For binding

experiments, GST or GST-tagged TMED10$^{1-130}$ at around the same abundance (~ 5 pmol) was pre-incubated at 4 °C for 30 min in a total volume of 450 μl binding buffer (20 mM Hepes, pH 7.2, 250 mM sorbitol, 70 mM KOAc, 1 mM $Mg(OAc)_2$, and 1 mg/ml bovine serum albumin). The coupled beads were blocked by a 40-min incubation at 4°C in 5 mM β-mercaptoethanol, 50 mM NaOAc, 0.5 M NaCl, pH 4.5, followed by washing steps and a 2 h incubation at 4°C in binding buffer. After incubation, 250 μl buffer containing around 5 μl beads conjugated with peptides was added to the reaction mixture at 4 °C for 80 min. The beads were washed five times by incubating with binding buffer containing 0.5M KOAc and 0.1% Triton without BSA for 1.5 min, followed by washing with binding buffer containing 0.1% Triton without BSA for 3 times. Then the beads were analyzed by immunoblot.

In vitro vesicle formation assay

In vitro vesicular release assays were performed as described previously (3, 4). Briefly, HeLa cells were un-transfected or transfected with control siRNA or siRNA against TMED10. Day 1 after transfection, cells were transfected with plasmids encoding RUSH-IGF2-HA. Day 2 after knockdown, HEK293T cells were permeabilized in 3 ml of ice-cold KOAc buffer (110 mM potassium acetate, 20 mM Hepes, pH 7.2, 2 mM magnesium acetate) containing 40 mg/ml digitonin on ice for 5 min. The semi-intact cells were then sedimented by centrifugation at 300 g for 3 min at 4 °C. The cell pellets were washed twice with 1 ml of KOAc buffer and resuspended in 100 μl of KOAc buffer. The budding assay was performed by incubating semi-intact cells (around 0.02 OD/reaction) with 2 mg/ml of rat liver cytosol in a 100 μl reaction mixture containing 200 mM GTP and an ATP regeneration system (40 mM creatine phosphate, 0.2 mg/ml of creatine phosphokinase, and 1 mM ATP) in the presence or absence of 0.5 mg of SAR1A (H79G). After incubation at 32 °C for 1 h, the reaction mixture was centrifuged at 14,000 g to remove cell debris and large membranes. The medium-speed supernatant was then centrifuged at 100,000 g to sediment small vesicles. The pellet fraction was then resuspended in 100 μl of 35% OptiPrep and overlaid with 700 μl of 30% OptiPrep and 30 μl of KOAc buffer. The samples were centrifuged at 55,000 rpm in a TLS55 rotor in a Beckman ultracentrifuge at 4 °C for 2 hr. After centrifugation, the top fraction was analyzed by SDS-PAGE and immunoblot.

The vesicle formation assay was then performed in a large scale using one 15 cm dish of WT HeLa cells or TMED10 KO HeLa cells to provide donor membranes. The vesicle fraction was then analyzed by label-free quantitative mass spectrometry using the procedure as previously described (5).

Vesicle immunoprecipitation (vesicle-IP) assay

One 10-cm dish of HeLa cells with or without transfection was used to provide semi-intact cells for the vesicle formation assay in each experimental group of the vesicle-IP assay. The semi-intact cells were incubated with 2 mg/ml of rat liver cytosol in a 1.8 ml reaction mixture containing 200 mM GTP and an ATP regeneration system at 32 °C for 1 h. The reaction mixture was centrifuged at 16900 g centrifugation

for 20 min. The medium-speed supernatant was then incubated with 30 µl Pierce anti-HA Magnetic beads at 4 °C overnight with rotation. The next day, the beads were collected with a magnetic stand. The supernatant was collected and centrifuged at 100K g in an S120AT3 (Hitachi) rotor at 4°C for 30 min to sediment vesicles as "flow-through". The beads were washed with ice-cold KOAc buffer containing 1 M, 0.5 M, and 0.11 M potassium acetate respectively, each for 3 times. The proteins in the flow-through fraction and in the on beads fraction were analyzed by immunoblot.

Sample preparation for the mass spectrometry analysis of the secreted proteins by C2C12 cells.

Undifferentiated C2C12 cells grown in 15 cm dishes were cultured with DMEM containing 20% FBS and 1% penicillin-streptomycin mix until the cells reached 80-90% confluence. Then C2C12 cells were transfected with siRNA against GFP or siRNA against mouse TMED10. Day1 after transfection, cells were washed five times with PBS and then cultured in DMEM containing 1% penicillin-streptomycin mix for 3 days. The culture medium was collected, and cell debris was removed by centrifugation. Subsequently, TCA precipitation was performed to precipitate the secreted proteins from the medium. The precipitated proteins were analyzed by SDS-PAGE and Coomassie Blue (Bio-SafeTM Coomassie-G250) staining. Subsequently, in-gel digestion and label-free mass spectrometry were performed as described (2).

SI References

1. Y. Guo, G. Zanetti, R. Schekman, A novel GTP-binding protein-adaptor protein complex responsible for export of Vangl2 from the trans Golgi network. *Elife* **2**, e00160 (2013).
2. X. Tang *et al.*, A SURF4-to-proteoglycan relay mechanism that mediates the sorting and secretion of a tagged variant of sonic hedgehog. *Proc Natl Acad Sci U S A* **119**, e2113991119 (2022).
3. X. Tang *et al.*, Molecular mechanisms that regulate export of the planar cell-polarity protein Frizzled-6 out of the endoplasmic reticulum. *J Biol Chem* 10.1074/jbc.RA120.012835 (2020).
4. X. Tang, F. Yang, Y. Guo, Cell-free Reconstitution of the Packaging of Cargo Proteins into Vesicles at the trans Golgi Network. *Bio Protoc* **10**, e3537 (2020).
5. Y. Huang *et al.*, An in vitro vesicle formation assay reveals cargo clients and factors that mediate vesicular trafficking. *Proc Natl Acad Sci U S A* **118** (2021).

Figure S1

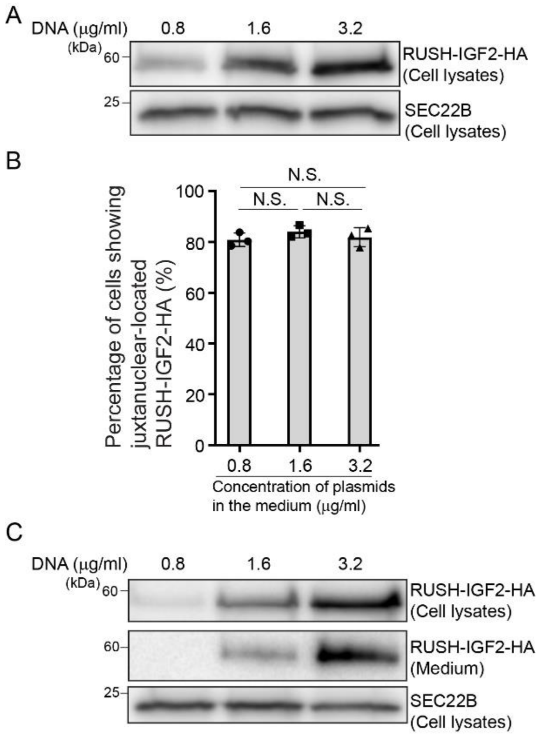

Figure S1. Analysis of trafficking of RUSH-IGF2-HA under different conditions of expression levels. HeLa cells were transfected with the indicated concentrations plasmids encoding RUSH-IGF2-HA. Day 1 after transfection, cells were pre-incubated with cycloheximide for 2 hr. Then cells were incubated with cycloheximide in the presence or absence of biotin for 20 min (B) or 2 hr (C). The level of RUSH-IGF2-HA and SEC22B in cell lysates from cells incubated in the absence of biotin were analyzed by immunoblot (A and C, Cell lysates). The percentage of cells showing juxtanuclear-localized RUSH-IGF2-HA 20 min after biotin treatment was quantified (B, mean ± S.D.; n = 3; >100 cells counted in each experiment). The level of RUSH-IGF2-HA in the medium 2h after biotin treatment was analyzed by immunoblot (C, RUSH-IGF2-HA, Medium). N.S., not significant.

Figure S2

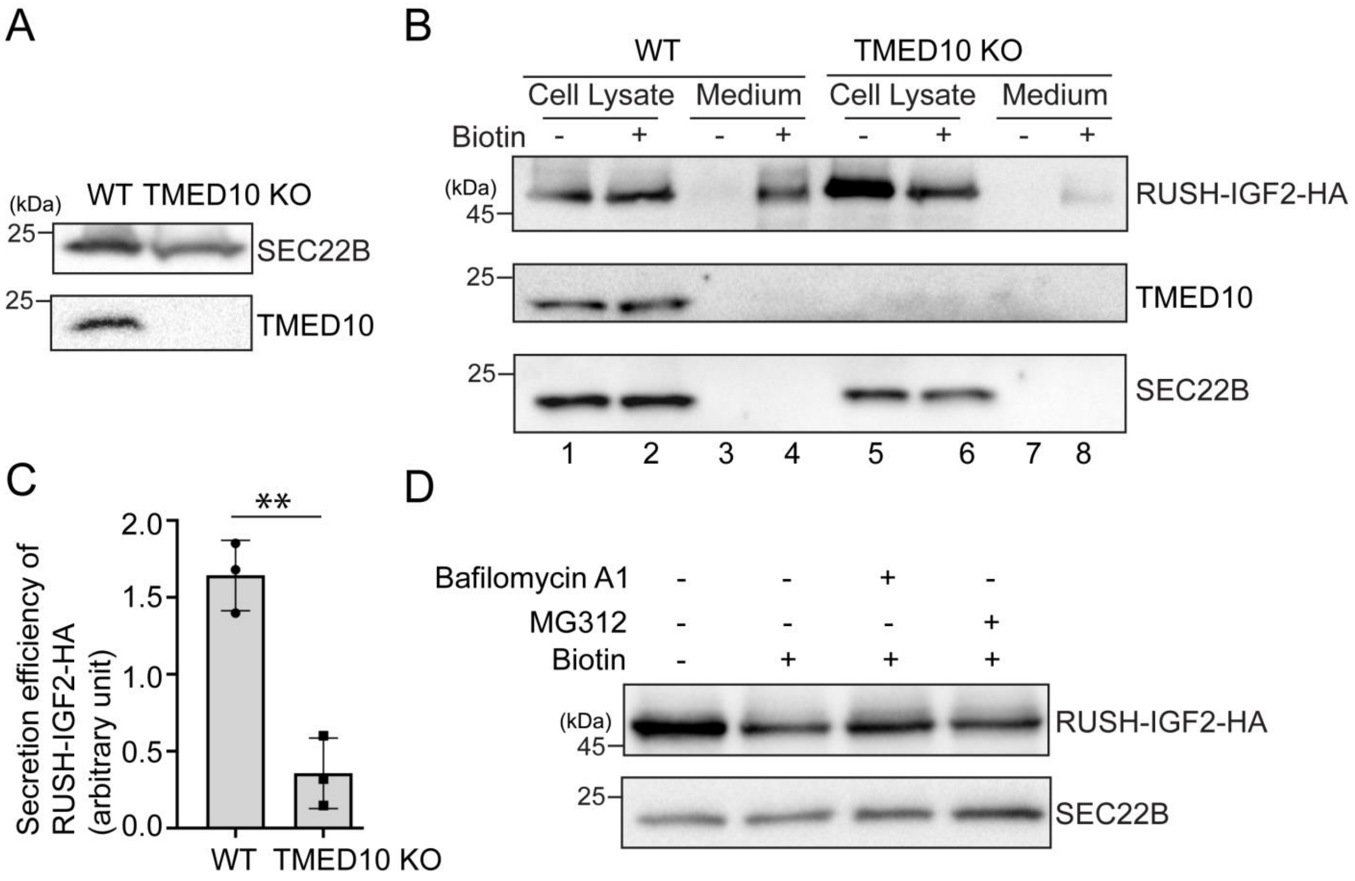

Figure S2. Knockout of TMED10 causes defects in secretion of IGF2. A. Cell lysates of WT or TMED10 KO HeLa cells were analyzed by immunoblotting with the indicated antibodies. **B.** WT or TMED10 KO HeLa cells were transiently transfected with RUSH-IGF2-HA. Day1 after transfection, cells were pre-incubated with cycloheximide for 2 hr. Then cells were incubated with biotin and cycloheximide for 2 hr. After biotin incubation, the level of RUSH-IGF2-HA in the medium and in cell lysates were analyzed by immunoblot. **C.** Quantification of the abundance of secreted IGF2 normalized to the abundance detected in the cell lysate group (mean ± S.D.; $n = 3$). In each replicated experiment, the value in each experimental group was normalized to the average value of the WT and TMED10 KO group. **, $p < 0.01$. **D.** TMED10 KO HeLa cells were transiently transfected with RUSH-IGF2-HA. Day1 after transfection, cells were pre-incubated with cycloheximide with/without 100 nM Bafilomycin A1 or 50 μM MG132 for 2 hr. Cells were incubated with biotin for 2hr. After incubation, the level of RUSH-IGF2-HA in cell lysates were analyzed by immunoblot.

Figure S3.

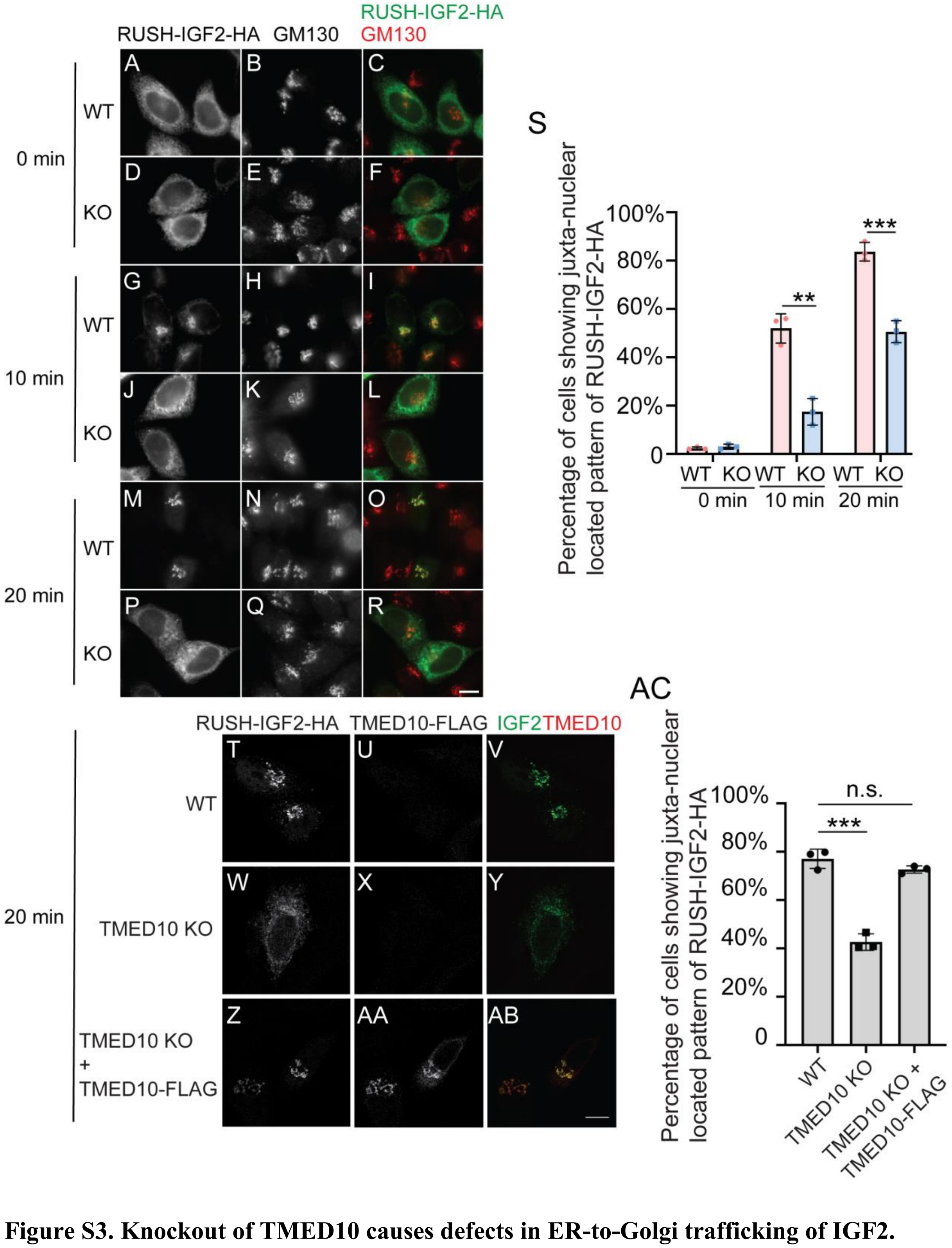

Figure S3. Knockout of TMED10 causes defects in ER-to-Golgi trafficking of IGF2.
A-R and T-AB. WT or TMED10 KO HeLa cells were transiently transfected with plasmids encoding the RUSH-IGF2-HA (A-R and T-Y) or co-transfected with plasmids encoding RUSH-IGF2-HA and TMED10-FLAG (Z-AB). Day 1 after transfection, cells were pre-incubated with cycloheximide for 2 hr. Then cells were incubated with biotin and cycloheximide at 37 °C for indicated time points and the localization of RUSH-IGF2-HA was analyzed by immunofluorescence. *Size Bar*, 10 μm. **S and AC.** The percentage of cells showing juxta-nuclear localization patterns of RUSH-IGF2-HA in each experimental group was quantified (mean ± S.D.; n = 3: >100 cells counted in each experiment). **, p<0.01; ***, p<0.001; n.s., not significant.

Figure S4

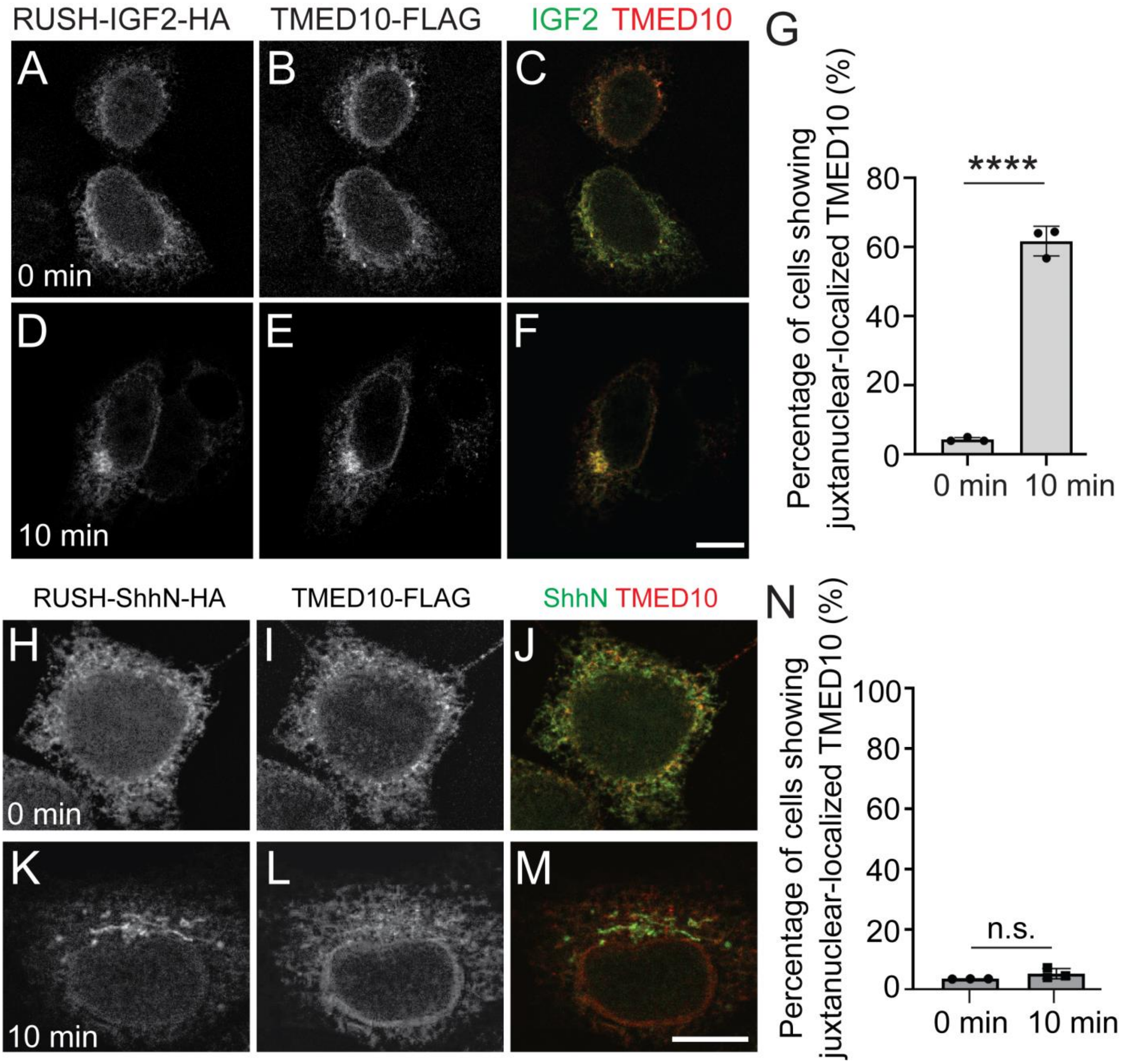

Figure S4. TMED10-FLAG traffics together with RUSH-IGF2-HA from the ER to the Golgi. A-F and H-M. HeLa cells were co-transfected with RUSH-IGF2-HA and TMED10-FLAG or co-transfected with RUHS-ShhN-HA and TMED10-FLAG. Day 1 after transfection, the localizations of the indicated proteins were analyzed after biotin treatment for 0 min (A-C, H-J) or 10 min (D-F, K-M). *Size Bar*, 10 µm. **G and N.** Quantification of the percentage of cells showing juxta-nuclear localization patterns of TMED10-FLAG in cells co-expressing TMED10-FLAG and RUSH-IGF2-HA or RUSH-ShhN-HA after biotin treatment for the indicated time (mean ± S.D.; n = 3; >100 cells counted in each experiment). ****, p<0.0001; n.s. not significant.

Figure S5

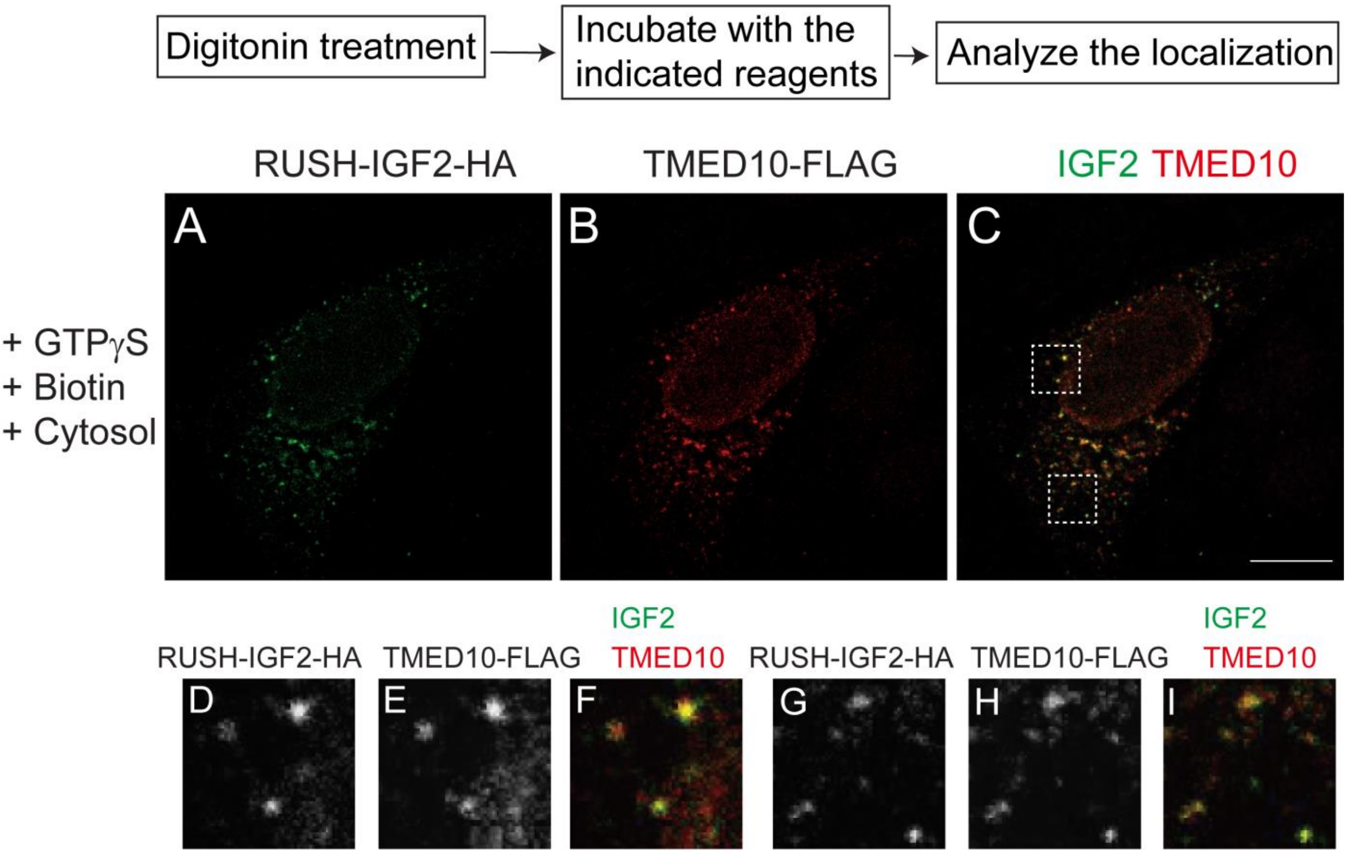

Figure S5. Analysis of the colocalization between RUSH-IGF2-HA and TMED10-FLAG using a permeabilized cell assay. A-C. HeLa cells were co-transfected with RUSH-IGF2-HA and TMED10-FLAG. Day 1 after transfection, cells were permeabilized by digitonin, then incubated with rat liver cytosol, biotin and GTPγS at 37°C for 15 min. After incubation, the localizations of the indicated proteins were analyzed by immunofluorescence. *Size Bar*, 10 μm. **D-I.** Magnified views of the indicated areas in panel C.

Figure S6

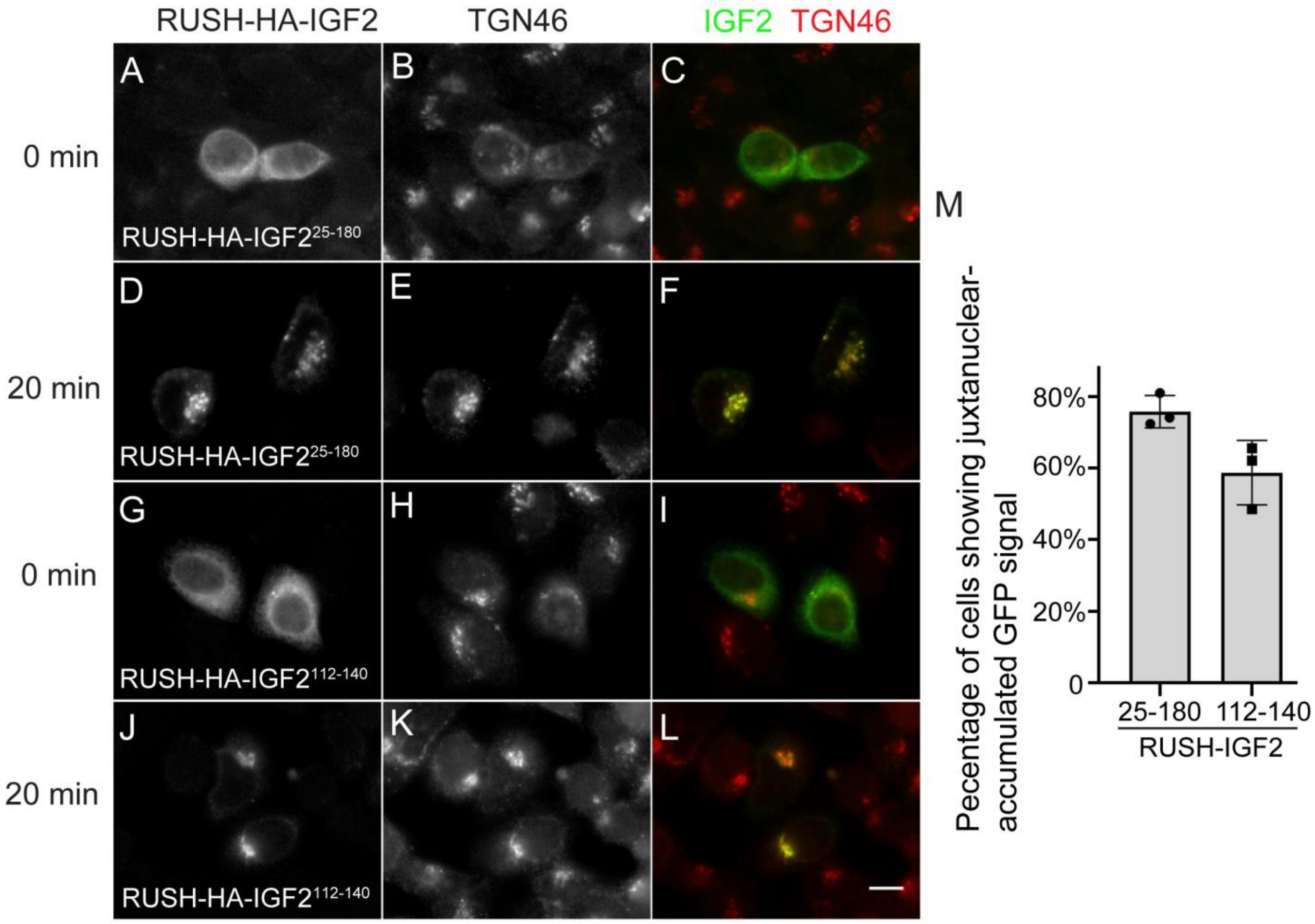

Figure S6. The residues 112-140 in IGF2 are sufficient to promote ER-to-Golgi trafficking. A-L. HeLa cells transfected with plasmids encoding the indicated RUSH constructs were incubated with biotin for the indicated period of time. The localizations of the indicated proteins were analyzed by immunofluorescence. *Size Bar*, 10 µm. **M.** Quantification of the percentage of cells showing juxtanuclear localization patterns of the RUSH construct 20 min after biotin treatment (mean ± S.D.; n = 3; >100 cells counted in each experiment).
